# Hot-electron transfer in quantum-dot heterojunction films

**DOI:** 10.1038/s41467-018-04623-9

**Published:** 2018-06-13

**Authors:** Gianluca Grimaldi, Ryan W. Crisp, Stephanie ten Brinck, Felipe Zapata, Michiko van Ouwendorp, Nicolas Renaud, Nicholas Kirkwood, Wiel H. Evers, Sachin Kinge, Ivan Infante, Laurens D. A. Siebbeles, Arjan J. Houtepen

**Affiliations:** 1 Optoelectronic Materials Section, Department of Chemical Engineering, Delft University of Technology, Van der Maasweg 9, 2629HZ Delft, The Netherlands; 2 Department of Theoretical Chemistry, Vrije Universiteit, 1081 HV Amsterdam, The Netherlands; 3 Netherlands eScience Center, Science Park 140, 1098 XG Amsterdam, The Netherlands; 4 Kavli Institute of Nanoscience, Delft University of Technology, Van der Maasweg 9, 2629 HZ Delft, The Netherlands; 5 Toyota Motor Europe, Materials Research and Development, Hoge Wei 33, B-1930 Zaventem, Belgium

## Abstract

Thermalization losses limit the photon-to-power conversion of solar cells at the high-energy side of the solar spectrum, as electrons quickly lose their energy relaxing to the band edge. Hot-electron transfer could reduce these losses. Here, we demonstrate fast and efficient hot-electron transfer between lead selenide and cadmium selenide quantum dots assembled in a quantum-dot heterojunction solid. In this system, the energy structure of the absorber material and of the electron extracting material can be easily tuned via a variation of quantum-dot size, allowing us to tailor the energetics of the transfer process for device applications. The efficiency of the transfer process increases with excitation energy as a result of the more favorable competition between hot-electron transfer and electron cooling. The experimental picture is supported by time-domain density functional theory calculations, showing that electron density is transferred from lead selenide to cadmium selenide quantum dots on the sub-picosecond timescale.

## Abstract (web summary)

Efficient use of high-energy, or “hot”, carriers could increase the efficiency of solar cells, provided efficient extraction of electrons at a specific energy. Here, the authors show the presence of hot-electron transfer between two quantum dot species, allowing facile optimization of the extraction energy.

## Introduction

Semiconductor quantum-dots (QDs) have drawn considerable interest due to their low-cost solution-based synthesis and unique photophysics, controllably bridging the divide between molecular and bulk material properties^1,2^. As the size of a semiconductor crystal is decreased to below the bulk exciton Bohr-radius, quantum-confinement starts breaking the continuous band-structure into discrete electronic levels. It has been suggested that the sparse density of states in QDs could slow electron cooling^3^, as single phonon emission does not suffice to bridge the energy between levels and slower multi-phonon emission events are needed to dissipate the electron energy. Such a “phonon-bottleneck” would enable making use of high energy (“hot”) carriers before they thermalize, for instance via carrier multiplication (CM)^4^ or hot-electron transfer (HET), provided appropriate quenching of surface related relaxation channels^3,5,6^. In addition to posing an interesting scientific problem, these processes may also find applications in solar energy conversion via suppression of thermal losses. Hot-electron solar cells in particular can theoretically enhance the maximum power conversion efficiency of solar cells from 33 to 66%.^7^

In practice, experimental evidence concerning slowing of carrier cooling in QDs remains scattered^3,8^ and cooling rates are usually high^9^. At the same time, CM and HET have been demonstrated using QDs. The interplay of cooling and CM or HET in nanostructure remains largely not well understood.

The HET process involves high-energy carriers, transferring between different species before thermalizing, and occurs in any materials where electron transfer outcompetes cooling. In recent years HET has been demonstrated to occur from QDs to metal-oxides^10,11^, acceptor molecules^12^ and localized surface states^13–15^. However, harvesting of hot-electrons to increase solar cell efficiency requires careful choice of both the absorber material band-gap and the energy of the extraction level. The latter is difficult to control in the previously reported hot-electron acceptors, requiring a change in the materials used for extraction.

In this work, we demonstrate ultrafast HET across PbSe-CdSe QD-heterostructures in QD heterojunction (QDHJ) films coupled by molecular linkers. We demonstrate that HET occurs in these QD HJs with an efficiency that increases as the excitation photon energy increases. Our results suggest that the facile control over the energetics of QDHJs can be used to spectrally tune photon absorption and electron injection without requiring changes in material composition.

## Results

### Films characterization

**Fig. 1:**
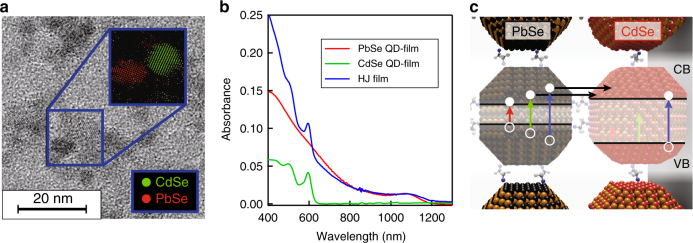
Film properties. **a** High-resolution transmission electron microscope (TEM) image showing PbSe and CdSe quantum dots (QDs) drop-casted on a TEM grid and subsequently treated with 1,2-ethanedithiol (EDT). The inset displays the result of a Fourier bandpass analysis of the image, distinguishing the two QD materials via their atomic lattices, and showing close proximity between the two components. **b** Linear absorption spectra of the combined PbSe-CdSe QDHJ film and of the single material films. **c** Schematic of the energy alignment in the system

QD heterojunction films were prepared by depositing alternating layers of PbSe and CdSe QDs on a quartz substrate (Supplementary Note 1, Supplementary Fig. 1-2), and exchanging the insulating ligands on the surface of the QDs with short conductive linkers^16,17^. The spectroscopic investigation focused on a sample fabricated with 1,2-ethanedithiol (EDT) linkers, while similar results are obtained treating each layer with 1,2-ethanediamine (EDA) (Supplementary Note 2, Supplementary Fig. 3-4). Figure 1a shows a transmission electron microscope (TEM) image of a reference film prepared with a single cycle of PbSe and CdSe QD deposition, displaying close proximity between the different QD components. The optical absorption spectra of the QDHJ film and of the reference single-material films are shown in Fig. 1b.

The energy alignment was investigated via spectro-electrochemical measurements on the QDHJ films (Supplementary Note 3, Supplementary Fig. 5). A type I alignment with a 1S energy level offset of 0.25 eV was found, in agreement with trends reported in literature^18^. This small conduction band offset, in conjunction with a significant difference in band gaps, allows selective study of the transfer of hot-electrons from PbSe to CdSe QDs. As shown schematically in Fig. 1c, upon low-energy photoexcitation (red and green lines) PbSe QDs are selectively photoexcited, while higher energy light is required to also photoexcite CdSe QDs (blue line). Excitation above the PbSe band gap may provide the electron with sufficient energy to transfer to the CdSe QD, while the hole is prevented from transferring by the large valence band offset.

### Hot-electron transfer probed by transient absorption spectroscopy

Transient absorption (TA) spectroscopy can be used to detect and quantify electron transfer between the two QD species. Upon selective photoexcitation of the PbSe QDs, electrons transferring to the CdSe QDs induce a decrease in the 1S CdSe absorption, commonly known as an absorption bleach, due to state filling and stimulated emission^19^. The differential absorbance Δ*A*_1S_ is proportional to the number of electrons in the CdSe 1S state, while holes contribute negligibly, on account of the higher degeneracy of hole states^20–22^. The CdSe Δ*A*_1S_ signal can then be used to quantify the number of electrons transferring to the initially unoccupied CdSe QDs.

**Fig. 2:**
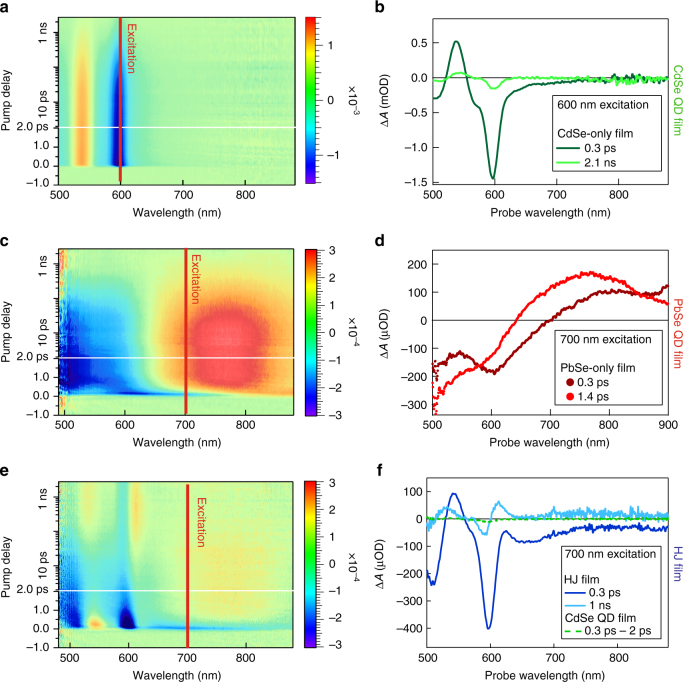
Transient absorption on QD films. **a** Color map showing the differential absorbance of a film composed of 4.5 nm CdSe QDs, excited at 600 nm with a fluence of 1.27 × 10^13^ photons/cm^2^ per pulse (0.07 excitons per CdSe QD). **b** Spectral cuts of (**a**), showing the TA response for two delay times after photoexcitation. **c** Color map showing the differential absorbance of a film composed of 2.3 nm PbSe QDs, excited at 700 nm with a fluence of 9.84 × 10^13^ photons/cm^2^ per pulse (0.39 excitons per PbSe QD). **d** Spectral cuts of (**c**). **e** Color map showing the differential absorbance of a QDHJ film, composed of 4.5 nm CdSe QDs and 2.3 nm PbSe QDs. The film is excited at 700 nm with a fluence of 1.19 × 10^14^ photons/cm^2^ per pulse (0.36 excitons per PbSe QD), closely resembling the excitation conditions of the PbSe QD film. **f** Spectral cuts of (**e**), showing the comparison between the TA response of the HJ film (dark and light blue) and the response of the CdSe QD film excited with the same conditions; i.e., 700 nm excitation with a fluence of 1.19 × 10^14^ photons/cm^2^ per pulse

In order to identify PbSe and CdSe QD contributions to the TA response of QDHJ films in the CdSe 1S spectral range, we characterized the response of films composed of the individual components. Figure 2a, b shows a TA measurement on an EDT-treated 4.5 nm CdSe QD film, whose absorption spectrum is shown in Fig. 1b, excited at 600 nm, clearly showing an absorption bleach at the CdSe 1S energy. The shape of the feature is maintained throughout the 3 ns window of the measurement, decreasing in amplitude as a result of charge carrier recombination (radiative and non-radiative).

Figure 2c, d shows the result of a TA measurement on EDT-treated 2.3 nm PbSe QD film, excited at 700 nm. At wavelengths in the visible, absorbance changes are characterized by the presence of a broad increase in absorbance (650–900 nm) and by an absorption bleach at the high-energy side of the probe window (500–650 nm). The photo-induced absorption feature is always observed in TA measurements of PbSe QDs, and has been attributed to biexciton shifts of the absorption spectrum^23,24^, while the bleach feature is typically seen in transient absorption or spectro-electrochemical measurement on PbSe QD films^25^, but not on dispersions. The nature of this broad absorption bleach has yet to be identified.

The TA response of the combined PbSe-CdSe film, excited at 700 nm, i.e., below the CdSe bandgap, is shown in Fig. 2e, f. As can be noted from the early time TA signal, both sets of TA features present in the individual films can be seen in the combined film response. In particular, the negative peak at the CdSe bandgap is associated with bleaching of the CdSe 1S transition, implying the presence of electrons at the CdSe conduction band edge. The spectral cut of the TA measurement in Fig. 2f compares the TA response of the combined film (continuous line) with the TA response of the CdSe-only film (dotted line), both excited at 700 nm with identical incoming photon fluence, proving that the bleach of the CdSe 1S feature in absence of neighboring PbSe QDs is negligible. We conclude that, as CdSe QDs are not directly photoexcited, electrons are transferred from PbSe to CdSe QDs. The excitation energy dependence of the rise time of the CdSe 1S bleach feature is in line with the suggested pathway (Supplementary Note 4, Supplementary Fig. 6).

The bleach at the CdSe 1S energy quickly decreases in a few ps. Considering the type I energy level structure (Fig. 1c) it is to be expected that after HET electrons quickly transfer back to the PbSe QD. To verify that this back-transfer is efficient we performed a separate experiment where we excited the CdSe 1S feature directly and quantified the efficiency of electron transfer by monitoring the ingrowth of the absorption bleach at the PbSe QDs 1S position (Supplementary Note 5, Supplementary Fig. 7). In that case, we observe fast electron transfer from the CdSe to the PbSe QDs with a near unity efficiency. Thus we conclude that the fast decay of the CdSe 1S bleach after HET observed in Fig. 2e, f is due to back transfer to PbSe QDs.

On a longer timescale of 50–100 ps, an anti-symmetric negative–positive feature appears at the CdSe 1S position and remains constant throughout the 3 ns measurement time. A second antisymmetric feature appears around 520 nm. This type of TA feature, resembling the first derivative of the absorption spectrum, is usually associated with an electric field induced shift of the absorption spectrum^26,27^.

The PbSe-related induced absorption between 650 and 850 nm decreases with the same time-constant as the shift features increase. These observations suggest that as charges depopulate the CdSe and PbSe QDs core states, excited carriers accumulate in proximity of CdSe QDs, electrostatically influencing the QDs and shifting the energy of the 1S and 1P absorption features. We tentatively attribute this to carrier trapping at the surface of the CdSe and/or PbSe QDs. However, for the current discussion the important observation is that CdSe 1S_e_ states are populated after selective excitation of PbSe QDs, indicating ultrafast HET from PbSe to CdSe QDs.

**Fig. 3:**
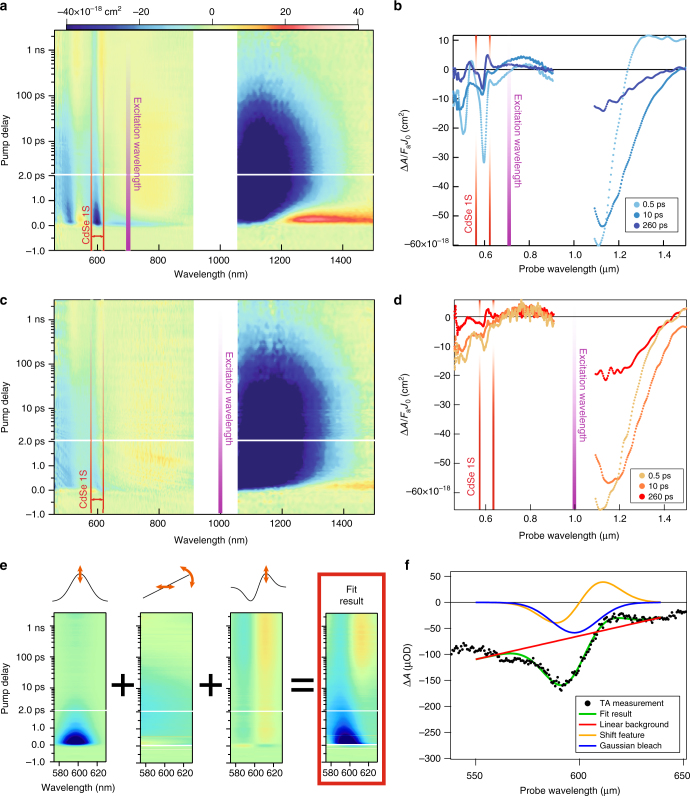
Heterojunction-film transient absorption response for different excitations and fitting method. **a** Transient absorption (TA) color map showing the differential absorbance per absorbed fluence of the combined PbSe-CdSe film, photoexcited at 700 nm with an absorbed fluence of 1.4 × 10^13^ photons/cm^2^ x pulse (0.36 excitons per PbSe QD). **b** Spectral slices of the color map 3a. **c** TA color map of the combined film photoexcited at 1000 nm with an absorbed fluence of 3.4 (3.0) × 10^13^ photons/cm^2^ x pulse in the visible (NIR) range (~0.07 excitons per PbSe QD). **d** Spectral cuts of color map 3c. **e** Color maps showing the different contributions to the fit of the TA response around the CdSe 1S feature; i.e. a Gaussian bleach, a linear background and a shift feature, as described in the text. The orange arrows in the schematics represent the 4 fit parameters (see Supplementary Note 6). **f** Spectral slice of a TA measurement showing the agreement between fit and experimental trace, together with the shape of the different contributions

We measured TA on the QDHJ film in both the visible (450–900 nm) and NIR (1150–1600 nm) spectral regions, varying the excitation energy to characterize the dependence of HET on the initial energy of the electron. Figure 3a–d shows the TA response of the QDHJ film normalized for the absorbed fluence (i.e., Δ*A*/*F*_a_*J*_0_, where *J*_0_ the incident photon fluence and *F*_a_ the fraction of absorbed light)^28^, excited at 700 and 1000 nm. For both excitations the NIR response is dominated by the absorption bleach of the lowest PbSe transition, while for 700 nm excitation an induced absorption feature is visible immediately after photoexcitation, which is related to a hot-carriers induced biexciton shift^23,24,29^. Besides the differences in the first picosecond, related to differences in carrier cooling, the NIR PbSe bleach remains largely the same for the two different excitations. In stark contrast, the CdSe TA features in the visible range differ significantly. For 1000 nm excitation, the CdSe 1S bleach is barely visible on top of the negative TA signal arising from PbSe QDs, indicating a much lower efficiency of hot-electron injection to the CdSe QDs compared to the measurement performed with 700 nm excitation wavelength.

### Hot-electron transfer efficiency

Figure 3 qualitatively shows that HET process depends on the energy of the absorbed photon. To quantify the HET efficiency and rate, the TA bleach of the CdSe 1S feature needs to be separated from other spectrally overlapping contributions, namely the slowly varying background, related to photoexcitation of PbSe QDs, and the shift feature prominent for *t* > 100 ps.

To separate the different contributions, we fitted the TA spectrum obtained at each delay-time with a superposition of a Gaussian bleach and a derivative-like shift feature, while the slowly varying PbSe contribution is approximated by a line^30^ (Supplementary Note 6-7, Supplementary Fig. 8). The color maps in Fig. 3e show the results of the fit to the TA measurement displayed in Fig. 3a, highlighting the behavior of the different contributions, while Fig. 3f displays the excellent match between the fitted function and the experimental data. This fitting method allows to extract the amplitude of the CdSe bleach component and to follow its time evolution. Furthermore, integrating the Gaussian profile over the entire 1S feature corrects for the effect of inhomogeneous broadening of the 1S feature and thus facilitates comparison between different samples. We will indicate this quantity as ΔA^*^ = ∫_1S_Δ*A*d*E*.

**Fig. 4:**
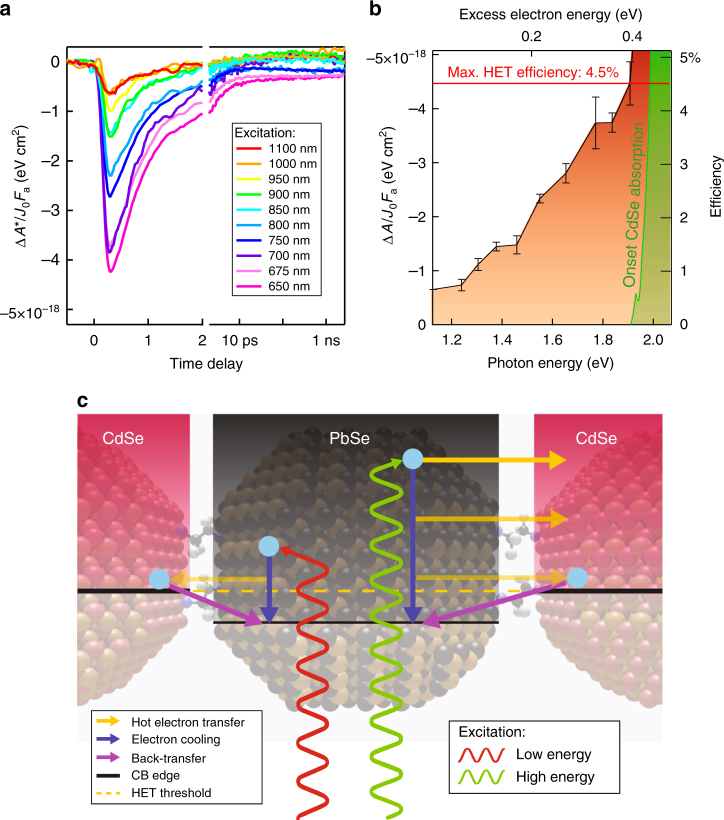
Excitation energy dependence of hot-electron transfer. **a** Fitted amplitude of the CdSe bleach component as a function of time, plotted for different excitation wavelengths. The plot shows an increase of the maximum bleach amplitude for shorter excitation wavelengths. **b** Plot of the bleach amplitude maxima as a function of excitation energy. The right axis shows the hot-electron transfer (HET) efficiency corresponding to each bleach value. Error bars are obtained from the standard deviation of the amplitude maxima, obtained from repeated measurements. **c** Schematics of the HET dynamics. Upon higher-energy excitation electrons have longer time available to transfer to CdSe QDs before cooling below the HET threshold than electrons excited at lower energy. In addition, transfer rates are expected to be larger for high-energy electrons, due to an increased amount of final CdSe states and more pronounced electron delocalization. The fast decrease of the CdSe bleach is attributed to back-transfer of electron from CdSe to PbSe QDs

We measured TA varying the excitation energy between 650 nm, the onset of the CdSe absorption (see Fig. 1b), and 1100 nm. Figure 4a shows the energy-integrated, absorbed fluence-rescaled differential absorbance of the CdSe 1S feature ΔA^*^/*F*_a_*J*_0_. The time dynamics shows a ~200 fs ingrowth of the signal (similar to the instrumental response limit), followed by decay with a *τ*_1/2_ of ~600 fs. A clear trend is observed in the excitation energy dependence of ΔA^*^, with a seven-fold signal increase between 1100 and 650 nm excitation. Sub-bandgap excitation of the CdSe QD reference film showed at least 20 times lower bleach signals than the combined film excited with equal photon fluence (Supplementary Note 8, Supplementary Fig. 9-10), thus demonstrating that direct CdSe excitation contributes negligibly to the combined film response.

To quantify the efficiency of the transfer process, we followed the procedure reported by Boehme et al.^28^ We first determine the bleach of the 1S absorption due to a single exciton by direct excitation in a CdSe QD reference film, i.e., the bleach cross-section $\sigma _b = \frac{{\left( {\Delta {{A}}^ \ast } \right)_{{\mathrm{max}}}{\kern 1pt} {\mathrm{ln}}\left( {10} \right)}}{{F_{\rm a}J_0}}$ (Supplementary Note 9). The efficiency of HET to CdSe QDs for selective excitation of PbSe QDs in a QDHJ film can then be expressed as:

1 $$\eta _{\mathrm {HET}} = \,\frac{{\left( \Delta{{A}^ \ast } \right)_{{\mathrm{max}}}}}{{F_{\mathrm {a}}J_0}}\,\frac{{{\mathrm{ln}}\left( {10} \right)}}{{\sigma _{\rm b}}}$$

where (ΔA^*^)_max_ is the peak of the integrated differential absorbance.

Figure 4b shows that the estimated HET efficiency increases as a function of excitation energy, a behavior recently observed for HET from QDs to molecular acceptors^12^. Exciting the system just below the CdSe absorption onset leads to a HET efficiency of 4.5%. For higher-energy excitations, electron injection from PbSe to CdSe QDs is mixed with the contribution stemming from direct photoexcitation of the CdSe QDs, which represents the dominant contribution to the CdSe bleach.

The observed trend in the electron injection efficiency can be understood within the HET picture schematically depicted in Fig. 4c. Electrons excited high in the manifold of PbSe conduction band states lose their energy quickly. When the energy of the electron drops below the CdSe QD 1S state, transfer is no longer possible. This implies that higher energy electrons have a longer time-window to undergo HET. At the same time the increase of the QD density of states as a function of electron energy leads to a higher number of available CdSe states, increasing the rate of electron transfer.

In these experiments, only electrons in the CdSe QD 1S_e_ level contribute to the bleach, while undetected higher-energy electrons can back-transfer to the PbSe QD component before reaching the conduction band-edge. Therefore, the highest extracted HET efficiency of 4.5% represents a lower limit for the real HET efficiency. Furthermore, higher transfer efficiencies may be achieved by further increasing the initial electron energy, although direct absorption from CdSe QDs prevents us from determining the transfer efficiencies at higher excitation energies.

### Rate-equation modeling

**Fig. 5:**
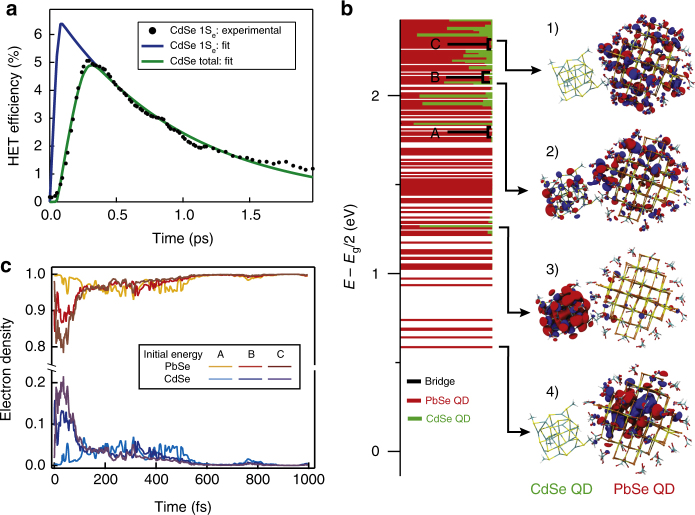
Modeling of hot-electron transfer. **a** Fit of the experimentally determined hot-electron transfer (HET) efficiency (black) with the rate-equation model discussed in the main text. The blue curve shows the fitted efficiency of charge injection in the CdSe 1S_e_ level, while the green curve indicates the total fraction of electrons injected in any CdSe state. The TA measurement is performed exciting the HJ film with 650 nm laser light, with a fluence of 4.14 × 10^12^ photons/(cm^2^ pulse). **b**, Computed electronic structure at the DFT/PBE level of theory of the PbSe-CdSe model system, which is composed of a PbSe QD and a CdSe QD coupled by a EDT molecule (bridge). The horizontal bars specify what fraction of each molecular orbital is localized on the PbSe QD (red), on the CdSe QD (green) and on the bridge (black). The contribution of the EDT bridge is almost negligible. The figure also shows the molecular orbital plots associated with 1) a high-energy state mostly localized on PbSe, 2) a high-energy state delocalized over both CdSe and PbSe, 3) the CdSe 1S_e_ state, and 4) the PbSe 1S_e_ state. **c** NAMD simulations illustrating the electron dynamics started from three different initial conditions indicated in (**b**) as A, B, and C. Electrons are rapidly injected into the CdSe QD, and transfer back on a longer timescale. The data show an increase in the maximum transferred electron density for initial states with higher energy

To obtain an estimate of the rate associated with the HET process from the HET efficiency, it is required to model the competition between HET and electron cooling. We employed a very simple rate-equation model, describing the conduction bands of the two materials as a discrete set of levels with equal energy spacing and electron transfer rates that are constant in energy and equal for forward and back transfer (Supplementary Note 10, Supplementary Fig. 11). The model was fitted to the dynamics of the CdSe 1S_e_ population in the QDHJ film, with the transfer rate and the energy-loss rates in the two materials as fitting parameters. Figure 5a shows the experimentally determined CdSe 1S_e_ population for 675 nm excitation (black), together with the fit of the population with the rate-equation model (blue). An electron transfer rate of 1.1 per ps and energy-loss rates of 2.2 and 0.5 eV/ps for PbSe and CdSe, respectively, are extracted from the fit. Figure 5a also shows the comparison between the fractional CdSe 1S_e_ population and the total HET efficiency (green), accounting for the charges that back-transfer before reaching the lowest CdSe excited state. In this way, a total HET efficiency of 6.2% is found.

The estimated energy-loss rates are in agreement with values reported in the literature^9,30,31^. The electron transfer rate of 1.1 per ps is similar to rates observed for “cold” electron transfer between CdTe and CdSe nanocrystals^28^ and for electron transfer between differently sized PbSe QDs^32^. Despite the strong oversimplifications, the model is able to describe the kinetics of the experimental signal, yielding reasonable values for the cooling and transfer rates.

### DFT modeling

To verify the extracted efficiency and rate of HET and to test the physical description of the competition between HET, cooling, and back transfer we also modeled the QDHJ system with density functional theory (DFT) calculations on a system composed of a PbSe QD and CdSe QD, bound covalently by a EDT bridge. To achieve the experimentally reported type-I band alignment, we employed a ~1.2 nm CdSe QD passivated with methane-thiol ligands and a ~1.8 nm PbSe QD passivated with formate ligands. After geometrical relaxation, we analyzed the electronic structure of this system at the DFT/PBE level of theory^33,34^ using the CP2k code (Fig. 5b)^35^. In this representation, each line corresponds to a molecular orbital (MO), and the color of the line represents the contribution of each fragment to this MO: the PbSe fragment is depicted in red, CdSe in green, and the organic bridge in black.

At energies near the CB edge, the MOs are mostly localized on the PbSe QD, with the exception of the 1S_e_ state of CdSe appearing at an energy of ~0.7 eV above the overall conduction band edge. At higher excess energies (>1.2 eV) MO mixing occurs between the CdSe and PbSe states. Such overlap is associated with the small inter-dot distance provided by the EDT bridge, and is reduced if longer bridge molecules are used (Supplementary Fig. 12-13). On the right of Fig. 5b, four different molecular orbitals are depicted: (1) a PbSe-localized MO located near the CB edge, (2) the 1S_e_ CdSe state that shows little mixing with PbSe, (3) a delocalized MO at high excess energies, showing contribution of all three fragments, and (4) a PbSe-localized MO also present at high excess energies.

We then performed time-domain non-adiabatic molecular dynamic (NAMD) simulations (see computational details in Methods section "TDDFT calculations of a coupled PbSe QD–CdSe QD system") to analyze the electron-phonon relaxation dynamics. Because we are interested only in the electron relaxation, we fixed the hole at the VB edge, and we started our NAMD simulations at different excess electron energies: ~1.2 eV (denoted as A in Fig. 5b), ~1.5 eV (B), and ~1.8 eV (C). Figure 5c shows the results of the NAMD simulations. For all simulations, electron density is seen to transfer rapidly from the PbSe QD to the CdSe QD on a ~50 fs timescale. Afterwards, the electron density is transferred back to PbSe, and after about 600 fs, electrons are again fully localized on the PbSe QD. Figure 5c also shows that by starting the dynamics at higher energy in the CB, the fraction of electron density transferred to CdSe increases, in agreement with the experimental observations presented above. The maximum fraction of electron density transferred to the CdSe QDs is 20% in the TDDFT calculation. This number, which includes electrons in all CdSe QD levels, not just the 1S_e_ level, is somewhat higher than the experimentally extracted 6.2%. The fact that the theoretical efficiency peaks at 20% is simply due to the fact that the electron density spreads over both the PbSe and CdSe QD states before localizing fully at the PbSe QD in the 1S_e_ level.

The combination of the experimental observation of HET and the DFT calculations shows that HET between QDs can indeed take place on very short timescales provided that the QDs are strongly coupled and wave functions at high energy are significantly delocalized over both QDs. This strong coupling is evident in the calculated wave functions and, given the good match with the experimental results, likely also occurs in the experimental QDHJ film. Using longer ligands to space the QDs will slow down both the HET process and the back-recombination rate. Engineering the coupling could potentially lead to an optimum between HET and back transfer, as has been shown for electron transfer from molecular dyes to TiO_2_ in dye sensitized solar cells^36^.

The above experiments show that HET between QDs is fast and feasible. However, for applications that aim to make use of hot-carriers it is clear that a pathway away from the CdSe QD–PbSe QD interface must be provided to avoid back transfer and to allow extraction of the carriers.

In conclusion, we have demonstrated ultrafast hot-electron transfer between two different quantum dot species. A maximum of 4.5% was observed for the transfer efficiency just below the CdSe QD absorption onset. Exciting above this threshold should give higher transfer efficiencies. Electrons injected in CdSe QDs are quickly transferred back to PbSe QDs, due to the type-I energy alignment. TDDFT calculations confirm the presence of sub-ps electron transfer between the two QDs and the dependence of the HET efficiency on the initial electron energy.

An efficient hot-carrier solar cell requires selective contacts, allowing only carriers within a narrow energy range to be extracted^7^. Hence, in addition to efficient HET, hot-carrier solar cells require control over the energy levels of both the donor and the acceptor in the HET process. The QDHJ system allows such control over the energy level of both the donor and the acceptor and, as demonstrated here, may also show efficient HET, indicating QDs as a promising material candidate for both light-absorbing and hot-electron extracting materials in HET solar cells.

## Methods

### Synthesis of CdSe quantum dots

CdSe QDs were obtained via a hot-injection synthesis method, adapted from van Embden et al.^37^. The QDs were synthetized by swift injection of a Se-precursor solution in a hot Cd-precursor solution held at 260° and kept under nitrogen atmosphere, followed by multiple injections of both solutions to sustain further QD growth. The Se injection solution was obtained dissolving 0.327 g Se powder in a solution of 2.5 g trioctylphosphine (TOP, tech grade 90%), 2.5 g 1-octadecene (ODE, tech grade 90%) and 6 g oleylamine (OAm, tech grade 70%), yielding a clear and slightly yellow solution, stored in a nitrogen-filled glovebox. The Se growth solution was obtained dissolving 0.25 g of Se powder in 1.55 g TOP in a nitrogen-filled glovebox, yielding a clear solution. The Cd-precursor growth solution was obtained adding 0.22 g CdO (99.999%), 0.970 g oleic acid (OA, 90%), and 6.23 g ODE to a 3-neck round-bottom flask (BPF) attached to a Schlenk line. The solution was degassed under vacuum (<1 mbar) for 1 h at 80°, it was heated to 260° under nitrogen atmosphere until it turned clear and then cooled back to room temperature. Oleylamine (1.13 mL, tech grade 70%) was added to the Cd-solution during cooling. The Cd growth solution was stored in a glovebox. Finally 0.22 g CdO, 3 g OA, and 30 g ODE were added to a 3-neck BPF flask, degassed under vacuum for 1 h at 80° and heated to 260° until the solution turned clear. The Se injection solution was loaded into a 24 mL syringe equipped with a 16G needle an quickly injected into the cadmium solution at 260°. The temperature of the reaction solution was allowed to recover to 250°, where it was held for QD growth. After 20 min, 2 mL of cadmium growth solution and 0.2 mL of selenium growth solution were added dropwise to the reaction. After 3 additions, one every 10 min, the reaction was allowed to proceed further for 10 min at 250°, then cooled at room temperature. The reaction solution was washed three times via QD precipitation, induced by the addition of acetone and centrifugation, and resuspension in toluene. After the last precipitation step, the QDs were resuspended in hexane and were stored in a glovebox.

### Synthesis of PbSe quantum dots

PbSe QDs were obtained via a hot-injection synthesis method, adapted from Steckel et al.^38^. The QDs were synthetized by swift injection of a Se-precursor solution in a hot Pb-precursor solution held at 120° and kept under nitrogen atmosphere. The Se injection solution was prepared dissolving 0.553 g Se powder in 19 mL TOP and adding 0.13 mL diphenylphosphine (DPP, 98%). The reaction solution was prepared adding to a 3-neck BPF flask 1.35 g PbO (99,999%), 17 mL ODE and 4 mL OA. The flask was connected to a Schlenk line, where the solution was degassed under vacuum (<1 mbar) for 1 h, then heated to 125° under nitrogen atmosphere, until it turned clear. The solution was further degassed under vacuum at 100° for half an hour, then heated back to 180° under nitrogen atmosphere. The Se injection solution was loaded into a 20 mL syringe equipped with a 16G needle, and quickly injected into the reaction solution. The solution temperature dropped to approximately 120° after injection. The reaction was allowed to proceed for 30 s, after which it was quenched by immersing the flash in water bath. The reaction solution was diluted in hexane, with addition of ethanol to induce QDs precipitation upon centrifuging. The washing procedure was repeated three times, then the QDs were resuspended in hexane and stored in a glovebox.

### TEM analysis

HR-TEM images were obtained from a JEOL-JEM 3200 FSC microscope. PbSe and CdSe QDs were deposited on a copper TEM grid covered with a 3 nm thick carbon supporting layer. A single dip-coating cycle was used for each QD material, to obtain roughly a monolayer coverage of the grid. A Fast Fourier Transform (FFT) analysis performed on the TEM images revealed the presence of two lattice periodicities. Applying a band-pass Fourier filter to the TEM image, in order to selectively display one of the two lattice spacing, we found that the smallest lattice spacing correspond to smaller QDs. Furthermore, FFT analysis of individual QDs revealed a square geometry for the reciprocal space points for the smallest spacing component, with a d-spacing of 3.08 nm, compatible with the expected values for a {200} PbSe plane. For the other component, a d-spacing of 3.7 was observed, which can be associated with a {111} plane in wurtzite CdSe. We conclude that both QD species are present on the TEM grids and that intimate contact is possible between QDs of the two materials.

### Film fabrication

The heterojunction films were fabricated via layer-by-layer growth with a mechanical dip-coater (DC Multi-8, Nima Technology), performed inside a nitrogen-filled glovebox. Each layer is obtained by dipping for 30 s a quartz substrate in a solution of QDs (PbSe or CdSe) in hexane, followed by 20 s drying outside the solution, 30 s dipping in a solution of the linker molecule (EDT or EDA) in acetonitrile. The concentration of the QD solution was 0.1 mM, determined from the linear absorption of the solutions and from the size-dependent extinction coefficient reported in literature^39,40^, while the ligand solution had a concentration of 10 mM for EDT and 1 M for EDA. The LbL procedure was repeated 14 times for the EDT capped film (12 for the EDA capped), yielding 7 (6) layers of each of the two QD materials. For each HJ film, two reference individual-QD films were fabricated, employing only one of the two QD solution and half of the total LbL cycles. Thickness measurements were performed scratching a QD film with a razor blade and measuring the depth of the scratch with a profilometer (DEKTAK 8, Veeco). A film thickness of 75 nm was measured for a HJ film fabricated with similar conditions as the EDT-capped HJ film measured in Transient Absorption, while the reference CdSe QD film had a thickness of 47 nm. These values correspond to a layer thickness of 1.6 QDs per layer for the CdSe QD component and 1.8 QDs per layer for the PbSe QD component.

### Transient absorption

Pump-probe TA measurements are performed on solid state samples placed inside an air-tight holder, loaded inside a nitrogen-filled glovebox. Two quartz windows on opposite sides of the holder allow to perform optical measurements on the sample. A Yb:KGW oscillator (Light Conversion, Pharos SP) is used to produce 180 fs pulses with a 1028 nm wavelength, at a 5 kHz frequency. The pump beam is obtained by sending the fundamental beam through an Optical Parametric Amplifier (OPA) equipped with a second harmonic module (Light Conversion, Orpheus), performing non-linear frequency mixing and producing an output beam whose wavelength can be tuned in the 310–1330 nm window. A small fraction of the fundamental beam power is used to produce a broadband probe spectrum (500–1600 nm), by supercontinuum generation in a sapphire crystal. The pump beam is transmitted through a mechanical chopper operating at 2.5 kHz, allowing one every two pump pulses to be transmitted. Pump and probe beam overlap at the sample position with a small relative angle (~8**°**), with a relative time delay controlled by an automated delay-stage. After transmission through the sample, the pump beam is dumped while the probe is collected at a detector (Ultrafast Systems, Helios). The differential absorbance is obtained via $\Delta A = {\mathrm{ln}}\left( {I_{\mathrm {on}}/I_{\mathrm {off}}} \right)$, where *I* is the light incident on the detector with either pump on or pump off. TA data are corrected for probe-chirp via a polynomial correction to the coherent artifact. Pump photon fluence was estimated by measuring with a thermopile sensor (Coherent, PS19Q) the pump beam transmission through a pinhole of 1 mm radius.

### TDDFT calculations of a coupled PbSe QD–CdSe QD system

To computationally investigate the electron injection between PbSe and CdSe QDs, we employed one of the most powerful approaches to study the electron-phonon relaxation dynamics: the non-adiabatic molecular dynamics (NA-MD) method. NA-MD combines a classical description of the nuclei motion and a time-dependent description of the electronic evolution, which includes quantum transition between electronically excited states. In this framework, electrons move in the potential energy surface of a single adiabatic electronic excited state, while the whole set of excited states, is computed “on-the-fly” at each step of the trajectory. Quantum transitions between different electronic states are evaluated stochastically using the fewest switches surface hopping (FSSH) method developed by Tully^41^. When implemented in the time-domain Kohn–Sham (TDKS)^42^, TDKS-FSSH can be used to study electronic transitions for large systems, whereas multi-electronic excited states are derived from one-electron transitions between the computed Kohn–Sham (KS) orbitals.

The time-dependent wave function of the system is calculated in the basis of KS orbitals by:

1 $$\left| {\psi \left( {x,t} \right)} \right\rangle = \mathop {\sum }\limits_{{k} = 1}^N c_{pk}\left(t \right)|\widetilde {\varphi _{p}}\left( {x;R} \right)\rangle$$

where $c_{pk}\left( t \right)$ are the time-dependent expansion coefficients and $\widetilde {{\mathrm{\varphi }}_{{p}}}\left( {{{x}};{{R}}} \right)$ is the adiabatic wave function representing the electronic excited state *p*. The electronic solution is obtained by solving the time-dependent Schrödinger equation:

2 $$i\hbar \frac{{\left. {\partial|\psi \left( {x,t} \right)} \right\rangle }}{{\partial t}} = H\left( {x,t} \right)|\psi \left( {x,t} \right)\rangle$$

By combining the two equations above, we obtain the time-dependent Schrödinger equation in the basis of the expansion coefficients:

3 $$i\hbar \frac{\partial }{{\partial t}}c_k\left( t \right) = \mathop {\sum }\limits_m^{N_e} c_m\left( t \right)\left( {\varepsilon _m\delta _{km} + d_{km}} \right)$$

where ${\mathrm{\varepsilon }}_{m}$ is the energy of the excited state *m* and $d_{km}$ is the time-derivative non-adiabatic coupling vector between states *k* and *m*, and can be reformulated as:

4 $$d_{km} = - i\hbar < \widetilde {\varphi _k}|\frac{\partial }{{\partial t}}\widetilde {\varphi _m} > $$

Typically, a standard electronic structure package provides the energies and the coefficients of the KS orbitals, while a separate module is needed to compute non-adiabatic couplings in (4). For this purpose, we have implemented a new module called QMflows-NAMD, which interfaces several quantum chemical codes with PYXAID, a program that describes the time evolution of electronically excited states as illustrated in equation (3)^43,44^. QMflows-NAMD is used to compute the molecular orbital coefficients, energies, and the nonadiabatic coupling elements between KS states at the DFT level of theory using the CP2k code. The non-adiabatic couplings are evaluated numerically using the Meek–Levine formula^45^. Additionally, a min-cost algorithm, implemented in QMflows-NAMD, is used to track the nature of each electronically excited state along the whole trajectory^46^. Finally, the non-adiabatic couplings and excited state energies are written on file in a format readable by PYXAID, which is then used to study the time evolution of the excited states. Here we employed the neglect the back-reaction approximation to decouple the electron dynamics from the nuclear dynamics, and ultimately using the ground state trajectory as the only meaningful one^47^. Such approximation has been demonstrated to be valid for large nanocrystals^48^.

To study the electron dynamics, we first relaxed the PbSe–CdSe system to its most stable structural configuration. We then performed an equilibration NVT dynamics using ab-initio DFT/PBE molecular dynamics simulation at 300 K using a velocity rescaling thermostat. Once the system had reached an equilibrium, we performed a production run with an NVE ensemble for 2 ps.

For each simulation, we used a single initial condition at *t*=0, and we solved stochastically between 7 and 14 hopping trajectories, depending on the number of available PbSe-localized states available in between CdSe-localized states at different excess energies.

### Data availability

The data that support the findings of this study are available on the 4TU repository, with the identifier 10.4121/uuid:89fabab0-9015-4e4f-a4da-67d286f2d15d.

## Electronic supplementary material

Supplementary Information

Peer Review File

## References

[1] Brus LE (1984). Electron-electron and electron-hole interactions in small semiconductor crystallites: the size dependence of the lowest excited electronic state. J. Chem. Phys..

[2] Banin U, Cao YW, Katz D, Millo O (1999). Identification of atomic-like electronic states in indium arsenide nanocrystal quantum dots. Nature.

[3] Pandey A, Guyot-Sionnest P (2008). Slow electron cooling in colloidal quantum dots. Science.

[4] Klimov VI (2006). Detailed-balance power conversion limits of nanocrystal-quantum-dot solar cells in the presence of carrier multiplication. Appl. Phys. Lett..

[5] Cooney RR (2007). Breaking the phonon bottleneck for holes in semiconductor quantum dots. Phys. Rev. Lett..

[6] Kambhampati P (2011). Hot exciton relaxation dynamics in semiconductor quantum dots: radiationless transitions on the nanoscale. J. Phys. Chem. C..

[7] Ross RT, Nozik AJ (1982). Efficiency of hot-carrier solar energy converters. J. Appl. Phys..

[8] Guyot-Sionnest P, Wehrenberg B, Yu D (2005). Intraband relaxation in CdSe nanocrystals and the strong influence of the surface ligands. J. Chem. Phys..

[9] Gao Y (2011). Enhanced hot-carrier cooling and ultrafast spectral diffusion in strongly coupled PbSe quantum-dot solids. Nano. Lett..

[10] Tisdale WA (2010). Hot-electron transfer from semiconductor nanocrystals. Science.

[11] Robel I, Kuno M, Kamat PV (2007). Size-dependent electron injection from excited CdSe quantum dots into TiO2 nanoparticles. J. Am. Chem. Soc..

[12] Li M (2017). Slow cooling and highly efficient extraction of hot carriers in colloidal perovskite nanocrystals. Nat. Commun..

[13] Pandey A, Guyot-Sionnest P (2010). Hot electron extraction from colloidal quantum dots. J. Phys. Chem. Lett..

[14] Sewall SL (2008). State-resolved studies of biexcitons and surface trapping dynamics in semiconductor quantum dots. J. Chem. Phys..

[15] Tyagi P, Kambhampati P (2011). False multiple exciton recombination and multiple exciton generation signals in semiconductor quantum dots arise from surface charge trapping. J. Chem. Phys..

[16] Law M (2008). Structural, optical, and electrical properties of PbSe nanocrystal solids treated thermally or with simple amines. J. Am. Chem. Soc..

[17] Luther JM (2008). Structural, optical and electrical properties of self-assembled films of PbSe nanocrystals treated with 1,2-ethanedithiol. ACS Nano.

[18] Boehme SC (2016). In situ spectroelectrochemical determination of energy levels and energy level offsets in quantum-dot heterojunctions. J. Phys. Chem. C..

[19] Klimov VI (2000). Optical nonlinearities and ultrafast carrier dynamics in semiconductor nanocrystals. J. Phys. Chem. B.

[20] Klimov VI, McBranch DW, Leatherdale CA, Bawendi MG (1999). Electron and hole relaxation pathways in semiconductor quantum dots. Phys. Rev. B.

[21] Sewall SL (2006). State-to-state exciton dynamics in semiconductor quantum dots. Phys. Rev. B.

[22] Fan F (2017). Continuous-wave lasing in colloidal quantum dot solids enabled by facet-selective epitaxy. Nature.

[23] Geiregat P (2014). Coulomb shifts upon exciton addition to photoexcited PbS colloidal quantum dots. J. Phys. Chem. C.

[24] Kambhampati P (2012). Multiexcitons in semiconductor nanocrystals: a platform for optoelectronics at high carrier concentration. J. Phys. Chem. Lett..

[25] Alimoradi Jazi M (2017). Transport properties of a two-dimensional PbSe square superstructure in an electrolyte-gated transistor. Nano. Lett..

[26] Empedocles SA, Bawendi MG (1997). Quantum-confined stark effect in single CdSe nanocrystallite quantum dots. Science.

[27] Trinh MT (2008). Nature of the second optical transition in PbSe nanocrystals. Nano. Lett..

[28] Boehme SC (2014). Electrochemical control over photoinduced electron transfer and trapping in CdSe-CdTe quantum-dot solids. ACS Nano.

[29] De Geyter B (2012). Broadband and picosecond intraband absorption in lead-based colloidal quantum dots. ACS Nano.

[30] Spoor FC (2016). Hole cooling is much faster than electron cooling in PbSe quantum dots. ACS Nano.

[31] Guyot-Sionnest P, Shim M, Matranga C, Hines M (1999). Intraband relaxation in CdSe quantum dots. Phys. Rev. B.

[32] Gao Y (2013). Disorder strongly enhances Auger recombination in conductive quantum-dot solids. Nat. Commun..

[33] Perdew JP, Burke K, Ernzerhof M (1996). Generalized gradient approximation made simple. Phys. Rev. Lett..

[34] Azpiroz JM, Ugalde JM, Infante I (2014). Benchmark assessment of density functional methods on group II-VI MX (M=Zn, Cd; X=S, Se, Te) quantum dots. J. Chem. Theory Comput..

[35] Hutter J, Iannuzzi M, Schiffmann F, VandeVondele J (2014). cp2k:atomistic simulations of condensed matter systems. Wiley Interdiscip. Rev..

[36] Kroeze JE (2006). Alkyl chain barriers for kinetic optimization in dye-sensitized solar cells. J. Am. Chem. Soc..

[37] van Embden J, Mulvaney P (2005). Nucleation and growth of CdSe nanocrystals in a binary ligand system. Langmuir.

[38] Steckel JS, Yen BK, Oertel DC, Bawendi MG (2006). On the mechanism of lead chalcogenide nanocrystal formation. J. Am. Chem. Soc..

[39] Moreels I (2007). Composition and size-dependent extinction coefficient of colloidal PbSe quantum dots. Chem. Mater..

[40] Jasieniak J (2009). Re-examination of the size-dependent absorption properties of CdSe quantum dots. J. Phys. Chem. C.

[41] Tully JC (1990). Molecular dynamics with electronic transitions. J. Chem. Phys..

[42] Craig CF, Duncan WR, Prezhdo OV (2005). Trajectory surface hopping in the time-dependent Kohn-Sham approach for electron-nuclear dynamics. Phys. Rev. Lett..

[43] Akimov AV, Prezhdo OV (2013). The PYXAID program for non-adiabatic molecular dynamics in condensed matter systems. J. Chem. Theory Comput..

[44] Akimov AV, Prezhdo OV (2014). Advanced capabilities of the PYXAID program: integration schemes, decoherence effects, multiexcitonic states, and field-matter interaction. J. Chem. Theory Comput..

[45] Meek GA, Levine BG (2014). Evaluation of the time-derivative coupling for accurate electronic state transition probabilities from numerical simulations. J. Phys. Chem. Lett..

[46] Fernandez-Alberti S (2012). Shishiodoshi unidirectional energy transfer mechanism in phenylene ethynylene dendrimers. J. Chem. Phys..

[47] Akimov, A. V. & Prezhdo, O. V. in *Encyclopedia of Nanotechnology, Theory of Nonadiabatic Electron Dynamics in Nanomaterials* (ed. Bhushan, B.) 1–20. (Springer, Netherlands, 2014).

[48] Akimov AV, Neukirch AJ, Prezhdo OV (2013). Theoretical insights into photoinduced charge transfer and catalysis at oxide interfaces. Chem. Rev..

