# Supplementary material for: Hot-electron transfer in quantum-dot heterojunction films
Source: Nat Commun. 2018 Jun 13;9:2310. doi: 10.1038/s41467-018-04623-9 (PMC5998019; doi:10.1038/s41467-018-04623-9)
Supplement: Supplementary file 1 — Supplementary Information [file 41467_2018_4623_MOESM1_ESM.docx]

**Hot-electron transfer in quantum-dot heterojunction films**

Supplementary Information

Gianluca Grimaldi^1^, Ryan W. Crisp^1^, Stephanie ten Brinck^2^, Felipe Zapata^2,3^, Michiko van Ouwendorp^1^, Nicolas Renaud^1^, Nicholas Kirkwood^1^, Wiel Evers^1,4^, Sachin Kinge^5^, Ivan Infante^2^, Laurens D. A. Siebbeles^1^, Arjan J. Houtepen^1^

**Affiliations:**

^1^Optoelectronic Materials Section, Department of Chemical Engineering, Delft University of Technology, Van der Maasweg 9, 2629 HZ Delft, The Netherlands

^2^Department of Theoretical Chemistry, Vrije Universiteit, 1081 HV Amsterdam, The Netherlands

^3^Netherlands eScience Center, Science Park 140, 1098 XG Amsterdam, The Netherlands

^4^Kavli Institute of Nanoscience, Delft University of Technology, Van der Maasweg 9, 2629 HZ Delft, The Netherlands

^5^Toyota Motor Europe, Materials Research & Development, Hoge Wei 33, B-1930 Zaventem, Belgium

**Supplementary Note 1: Fourier analysis of TEM images**

Supplementary Figure 1a shows a TEM image of a substrate dipped once in a solution of PbSe QDs (2.3 nm) and in a solution CdSe QDs (4.5nm), followed by ligand exchange with ethanedithiol (EDT) linkers. The small size of the PbSe QDs and the material contrast of the CdSe QDs did not allow us to resolve the two QD components with our TEM setup. Therefore, we performed a Fourier transform of the images, displayed in Supplementary Figure 1b, highlighting the presence of two resolvable lattice distances.

Radial integration of the Fourier transform, displayed in Supplementary Figure 1c, revealed the presence of two intensity peaks superimposed on a varying background. Fitting the two peaks with a Gaussian profile and a linear slope, approximately describing the background, yielded a d-spacing for the two features of 0.32 nm and 0.37 nm.


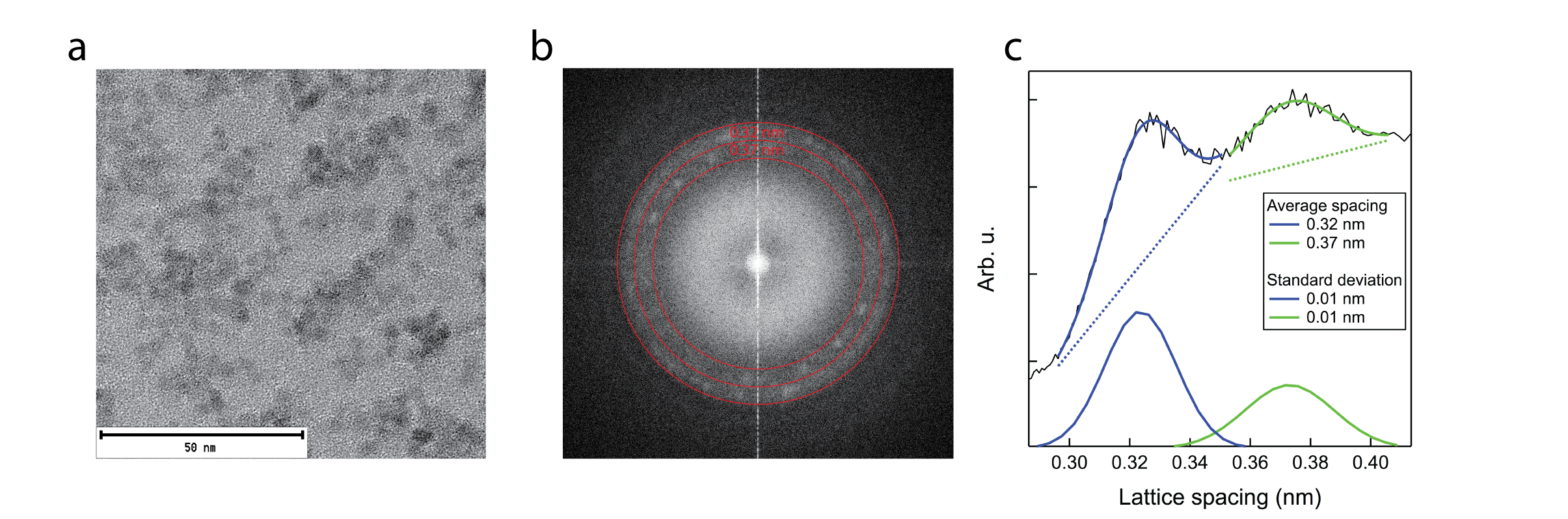


Supplementary Figure 1. Identification of the two quantum dot (QD) components via Transmission Electron Microscopy (TEM). a, TEM image of a sample fabricated dipcoating a TEM grid one time in a PbSe QD solution, one time in a CdSe QD solution, and ligand exchanging for EDT linkers between the cycles. b, Fourier transform of Supplementary Figure 1a, highlighting the presence of two rings of diffraction features. c, radial integration of Supplementary Figure 1b, fitted with a Gaussian feature and a line.

Supplementary Table 1. d-spacings for planes in rocksalt-PbSe.

| Rocksalt-PbSe Miller indices | d-spacing (nm) |
| --- | --- |
| {200} | 0.306 |
| {2-20} | 0.217 |
| {4-2-2} | 0.126 |
| {4-40} | 0.109 |

Supplementary Table 2. d-spacings for planes in wurtzite-CdSe.

| Wurtzite-CdSe Miller indices | d-spacing (nm) |
| --- | --- |
| {100} | 0.372 |
| {002} | 0.351 |
| {101} | 0.329 |
| {102} | 0.255 |

Applying a bandpass filter to the image, centered to one of the two periodicities, and taking the inverse Fourier transform of the image, we produce two images containing only the atomic features corresponding to the selected lattice spacing. Supplementary Figure 2a shows the superposition of the two images: the atomic feature corresponding to the 0.32 ± 0.02 nm periodicity (highlighted in red), those corresponding to the 0.38 ± 0.02 nm periodicity (in green). Supplementary Table 1 and Supplementary Table 2 show the calculated lattice spacing of different planes for rocksalt PbSe and wurtzite CdSe, respectively. It must be noted that, while the 0.38 nm can be unambiguously assigned to the periodicity of the {100} plane of wurtzite CdSe, the Fourier peak at 0.32 nm can contain contributions from both the CdSe {101} plane and the PbSe {200} plane. From Supplementary Figure 2a it can be seen how the QDs corresponding to the 0.32 nm periodicity have a smaller size than the other QDs, suggesting this periodicity can be attributed primarily to the presence of the smaller PbSe QDs. Applying the Fourier transform on the two neighbouring QDs shown in Figure 1a (red and green rectangles in Supplementary Figure 2a), produces the Fourier images in Supplementary Figure 2b and Supplementary Figure 2d. Supplementary Figure 2b corresponds to the QD with 0.32 nm spacing (red highlights) and clearly shows a square arrangement of the diffraction points. The d-spacing, shown by the intensity peaks in Supplementary Figure 2c, closely matches the predicted 0.306 nm spacing of {200} planes in PbSe. Similar analysis of the other QD (green highlights) reveals diffraction peaks with centered at 0.37 nm, closely matching the periodicity of {100} facets in wurtzite CdSe. Hence, we attribute the two QDs to the two different materials and note that they are in close proximity to each other but that they remain distinct particles.


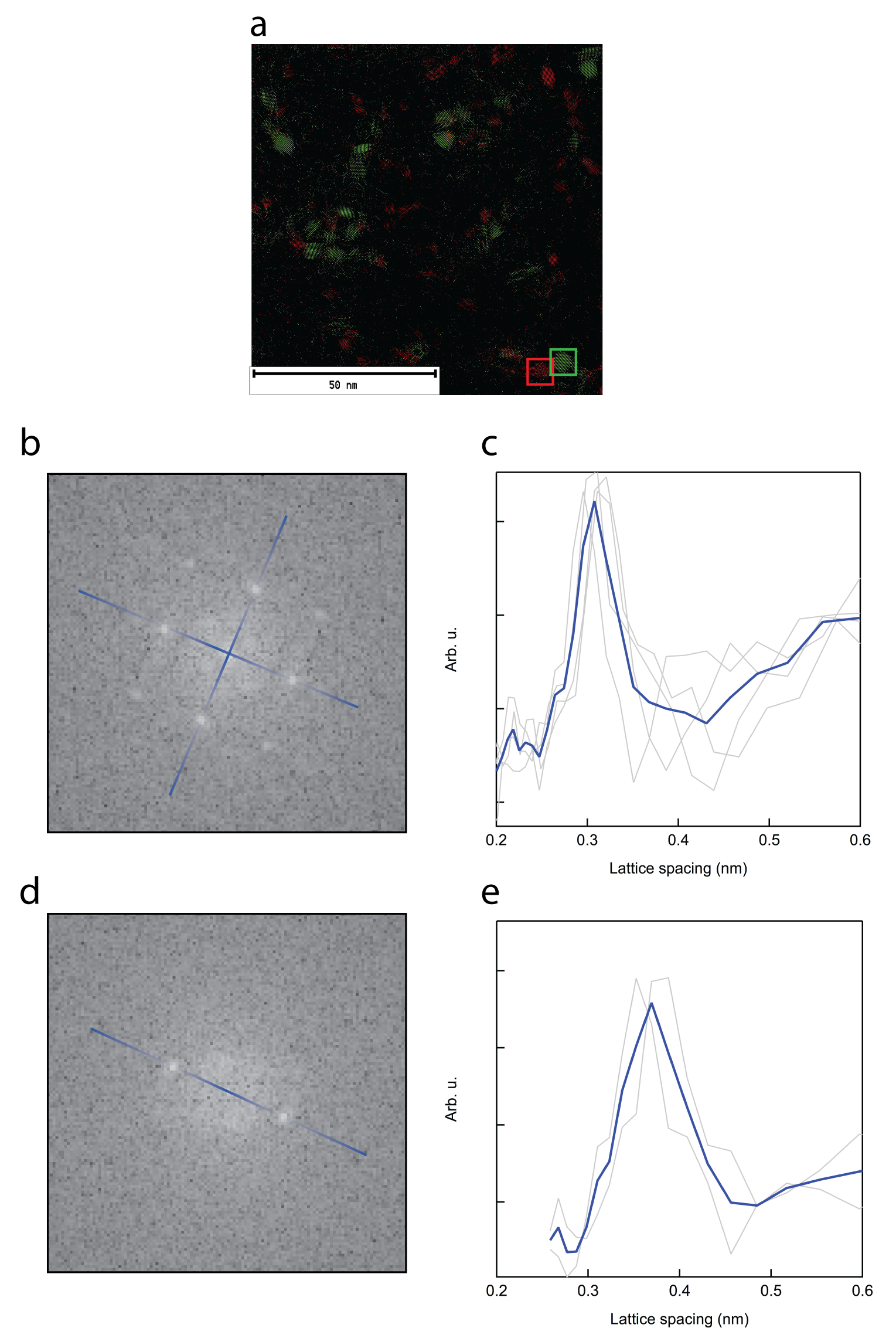


Supplementary Figure 2. Fourier analysis of neighboring QDs. a, superposition of two images obtained applying to the image in Supplementary Figure 1a two different Fourier bandpass filters, selecting the periodicity 0.32 ± 0.02 nm (red) and 0.38 ± 0.02 nm (green). b, Fourier transform of the portion of Supplementary Figure 2a contained in the green square. c, plot of the intensity of the diffraction features in Supplementary Figure 2b along the radial direction (blue lines). d, Fourier transform of the portion of Supplementary Figure 2a contained in the red square. e, plot of the intensity of the diffraction features in Supplementary Figure 2d along the radial direction (blue line).

**Supplementary Note 2: Transient Absorption results for an EDA-treated film**

This section shows the result of a TA investigation on the HJQD film treated during the layer-by-layer processing with ethanediamine (EDA) instead of EDT. The treatment is expected to reduce inter-particle distance *via* removal of the original ligands, leading to QD necking^4, 5^.


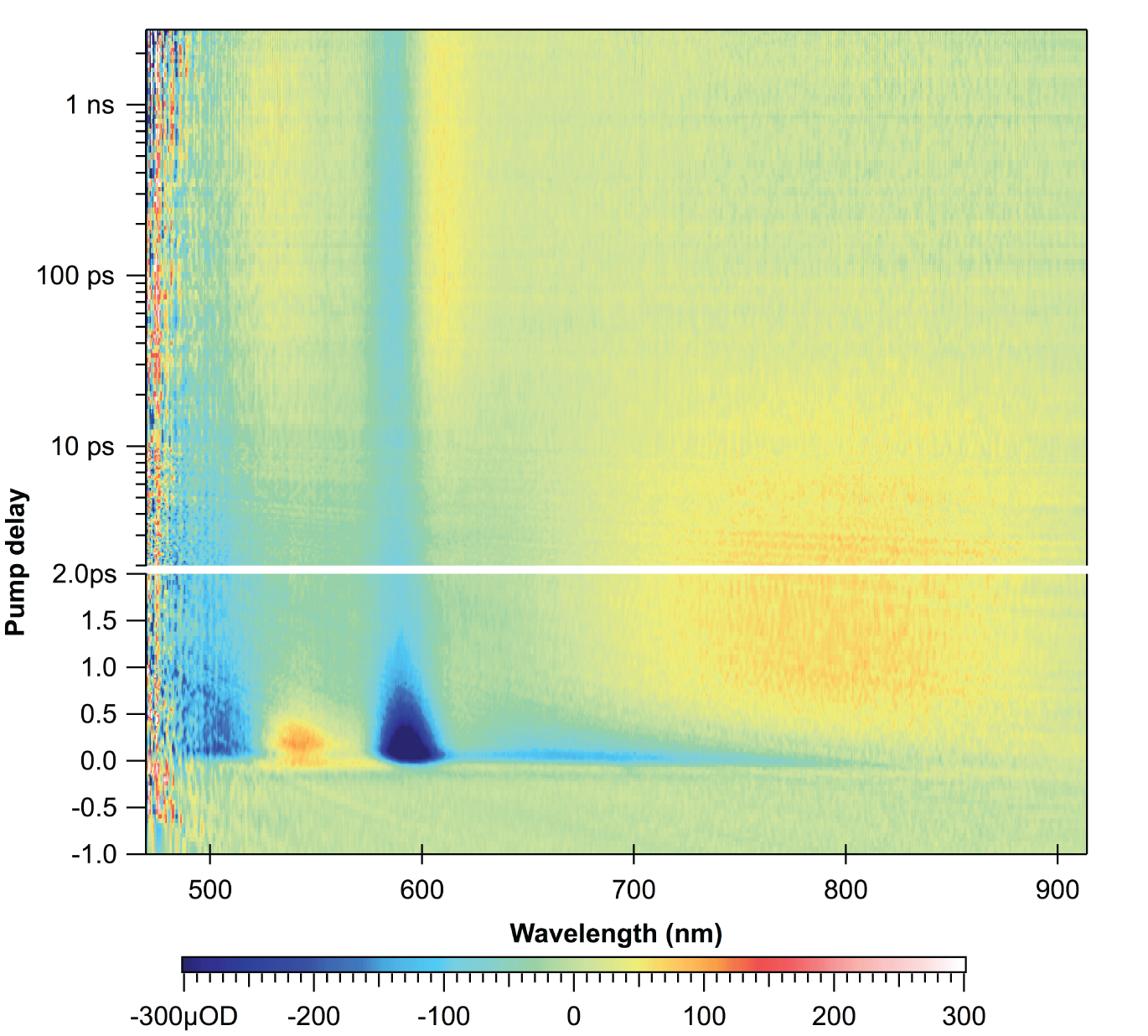


Supplementary Figure 3. TA response of an EDA-capped HJ film. Color map showing the differential absorbance of a HJQD film composed of 4.5 nm CdSe QDs and 2.3 nm PbSe QDs, treated with EDA. The film is excited at 700 nm with an absorbed fluence of 9.03$\boldsymbol{\cdot}$10^12^ photons/cm^2^ per pulse.


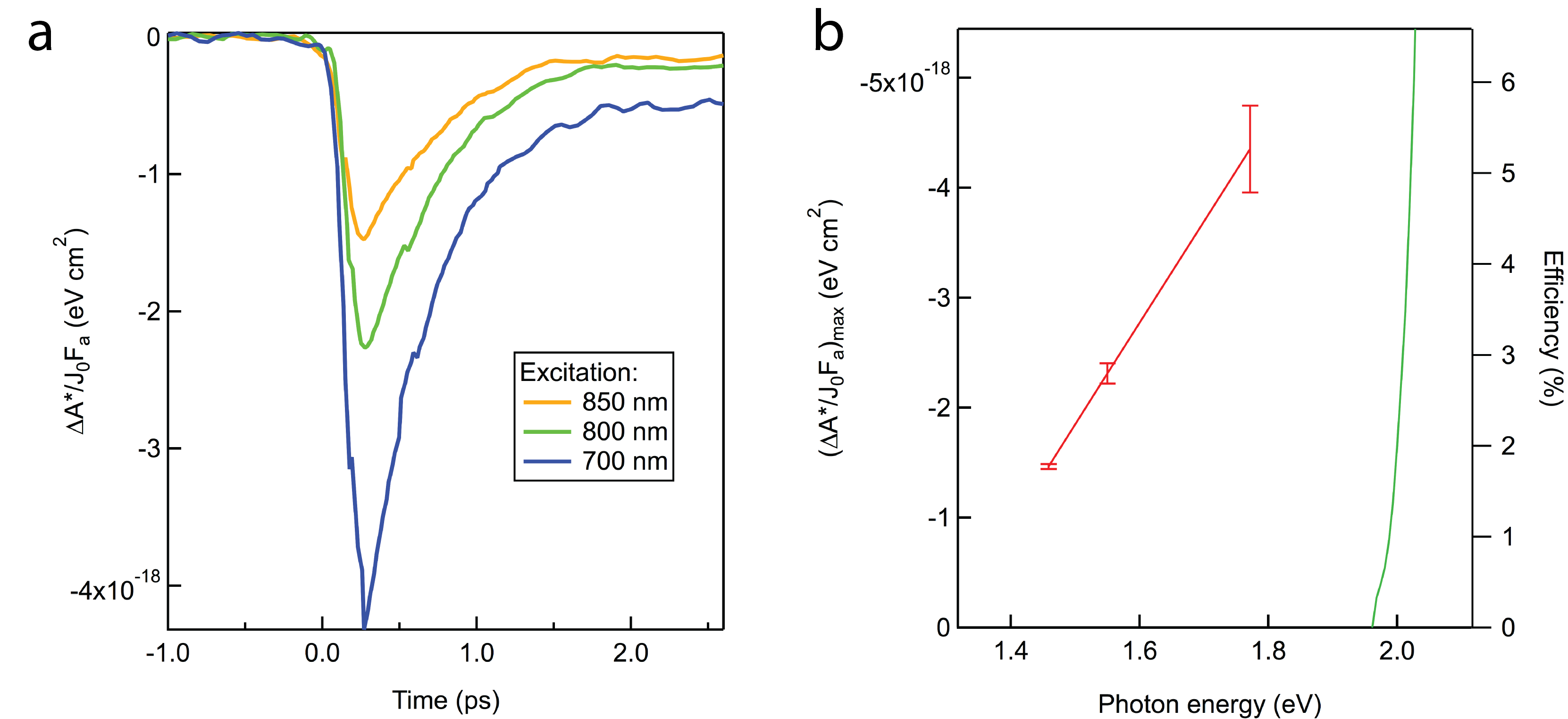


Supplementary Figure 4. Hot-electron transfer in an EDA-capped HJ film. a, fitted amplitude of the CdSe bleach component of the EDA-HJQD film, plotted for different excitation wavelengths. b, plot of the bleach amplitude maxima as a function of excitation energy. The right axis shows the HET efficiency corresponding to each bleach value. Error bars are obtained from the standard deviation of the amplitude maxima, obtained from repeated measurements. The green line indicates the onset of the CdSe QD absorption. Error bars are obtained from the standard deviation of the bleach amplitude maxima obtained in repeated measurements.

Supplementary Figure 3 shows a TA measurement performed on the EDA-treated HJQD film with 700 nm excitation, showing the same behavior observed for the EDT treated films: the appearance of a CdSe bleach upon photoexcitation, which decays in a sub-ps timescale; on a longer time-scale (~100 ps) a derivative-like feature appears at the CdSe bandgap energy. The traces in Supplementary Figure 4a show the normalized CdSe bleach of the EDA-HJQD film obtained fitting TA measurements performed with different excitation wavelengths. The initial value of the bleach increases for decreasing excitation wavelength, as observed for the EDT-treated films. HET efficiency values obtained for the measurements are shown in Supplementary Figure 4b, which are in line with the values obtained for the EDT-treated sample.

**Supplementary Note 3: Spectro-electrochemical determination of conduction-band offset in heterojunction film**

The HJ film used for the spectro-electrochemical measurements was prepared depositing alternating layers of the same QD materials used for the TA study, using a layer-by-layer dipcoating processing on an ITO substrate. During each deposition cycle the film is dipped into the QD solution, slowly extracted, leaving a layer of QDs on the substrate, and then ligand exchanged with ethanedithiol (EDT).

The spectro-electrochemical measurement was performed in a previously reported setup^1^. The sample was immersed in a LiClO_4_ electrolyte solution in acetonitrile, with a Ag wire pseudoreference electrode and a Pt counter electrode. The potential of the sample was controlled with a PGSTAT128N (Autolab) potentiostat, while changes in the sample absorbance were monitored simultaneously with a USB2000 (Ocean Optics) spectrometer (300-1000 nm) and a NIRQuest 256 (Ocean Optics) spectrometer (900-2500 nm). In the following text, reported values of the potential are relative to the potential of the Ag electrode.


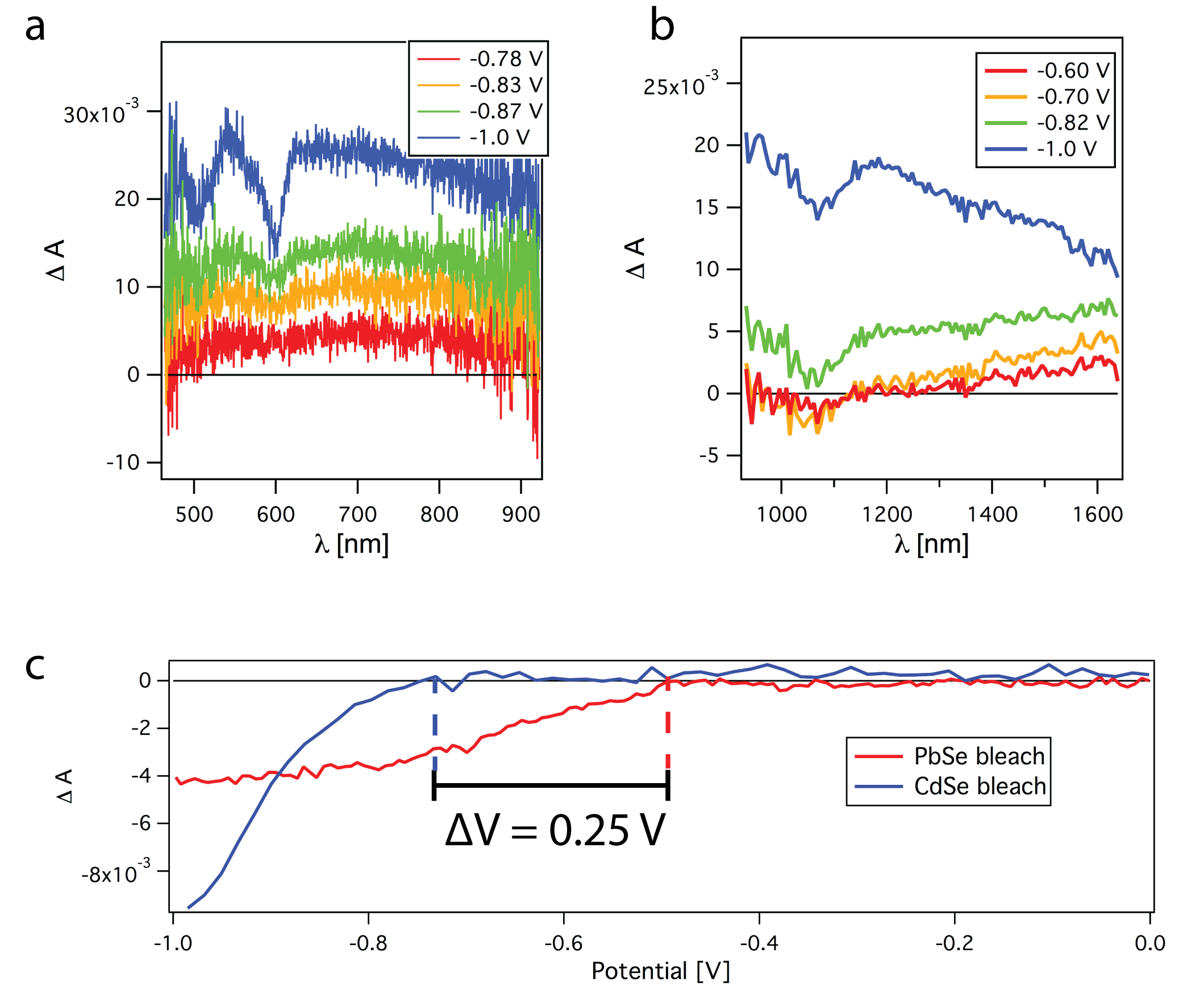


Supplementary Figure 5. Spectro-electrochemical determination of conduction band offset. a-b, differential absorbance of the QDHJ film as a function of the applied potential, for the visible (a) and NIR (b) spectral regions, highlighting the presence of two bleach features at the bandgap of the two QD materials. c, Amplitude of the Gaussian features fitted to the PbSe and CdSe bleach as a function of the applied potential.

The CV scan was performed with a scan rate of 20 mV/s, scanning from 0 to -1 V. Supplementary Figure 5a shows the absorbance changes of the QDHJ film in the visible range. No absorbance change is observed up to -0.7 V, after which a positive background starts increasing, remaining roughly constant over the visible range. Around -0.8 V a bleach feature appears on top of the positive background at the wavelength of CdSe 1S absorption (597 nm), indicating the injection of electrons in the conduction band of CdSe QDs. Supplementary Figure 5b shows the same potential scan, monitored in the NIR range. From ~-0.6 V a bleach feature appears at the wavelength of the PbSe 1S absorption (1068 nm), indicating electron injection to the conduction band of PbSe QDs. When the potential is lowered further, a broad induced absorption feature start increasing, and eventually dominates the signal for the lowest potentials. The spectral traces obtained from the two measurements where fitted at each applied potential, to separate the bleach contribution from the induced absorption background. The visible range data were fitted with the sum of a Gaussian term (CdSe bleach) and a constant background. The NIR signal was fitted with a Gaussian term (PbSe bleach) and a linear term, to empirically take into account the shape of the NIR background. Supplementary Figure 5c shows the amplitudes of the two Gaussian terms, allowing us to extract the potential difference between the onsets of the bleach features; i.e. $\Delta V=0.25 \text{V}$. The measured offset probably provides an upper limit for the actual conduction band offset, as additional energy is required to overcome charging effects.

**Supplementary Note 4: Rise dynamics of CdSe QDs bleach signal**

In order to quantify the rise time of the CdSe QDs bleach signal, we fitted the experimental bleach traces to the equation:

$$f\left( t \right)= A \left( 1+\mathrm{erf} \left[ \frac{t-t_{0}}{\sqrt{2}\cdot\sigma} \right] \right)$$

Where $\sigma$ is a coefficient indicating how quickly does the bleach signal rise. Supplementary Figure 6 shows a plot of $\sigma$ for different excitation wavelengths, indicating that the CdSe bleach rise time increases sharply going from direct CdSe QD excitation to a sub-CdSe bandgap excitation of the system. In this regime, the electron needs first to transfer from PbSe to CdSe QDs, and subsequently cool within the manifold of CdSe excited states, thus resulting in a longer build-up time for the bleach signal.

**
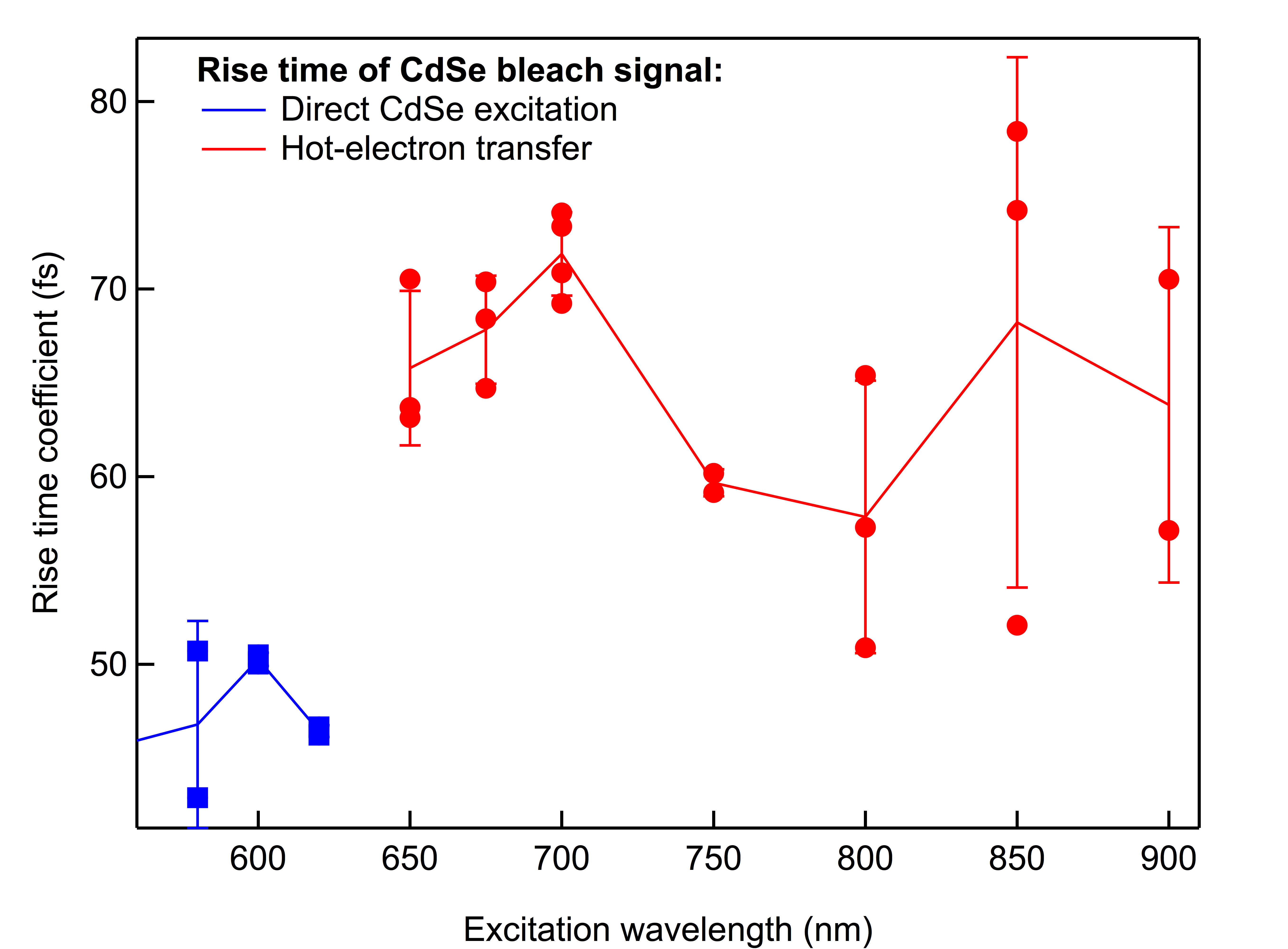
**

**Supplementary Figure 6.** **Rise time coefficient for the CdSe QD bleach signal as a function of excitation energies.** Going from direct excitation of CdSe QDs to the hot-electron transfer excitation regime leads to a sharp increase in the rise time, arising from the electron transfer pathway. Error bars represent the standard deviation of the rise time coefficients at each excitation wavelength, obtained from repeated measurements.

**Supplementary Note 5: Estimate of electron back-transfer from CdSe to PbSe QDs**

TA measurements on the QDHJ film show that the CdSe bleach decays rapidly after photoexcitation, for both below and above CdSe bandgap excitation, indicating that electrons leave the CdSe QDs. The measured type-I band alignment between the two QD materials (Supplementary Note 3) suggests electrons would favorably transfer from CdSe to PbSe QDs.

To confirm this relaxation pathway, we measured the TA response at the bandedge of the PbSe QD component, comparing the PbSe bleach measured with different excitation energies. Upon below CdSe bandgap excitation, only a small fraction of the excited electrons populate CdSe QDs (Fig. 4b). Conversely, above CdSe bandgap excitation results in more than 15% of the charges localizing on CdSe QDs, estimated with the same method employed to calculate HET efficiencies: rescaling the initial normalized bleach by the bleach cross section. Supplementary Figure 7 displays in as red dots the normalized PbSe bleach for below CdSe-bandgap excitation. Errors bars, when present, are obtained repeating the measurement with different excitation fluence and taking the standard deviation of the results. This procedure is allowed because of the linearity of the measured bleach signal with power. Comparing the average value of the PbSe bleach for below CdSe-bandgap excitation (red line) with the values obtained for above CdSe-bandgap excitation (green points), it is possible to notice that the difference is not statistically significant. Thus, within the accuracy of the measurement, initial excitation of a significant fraction of electrons in CdSe QDs doesn’t result in an appreciable change in the final electron population of the PbSe QDs. We then estimated the expected decrease in PbSe bleach in the absence of electron transfer, multiplying the average PbSe bleach with the fraction of charges initially photoexcited on PbSe QDs. The estimated bleach values (Supplementary Figure 7, blue dots) are significantly lower than what is experimentally observed. The result suggests near unity yield of the transfer process from CdSe to PbSe QDs.


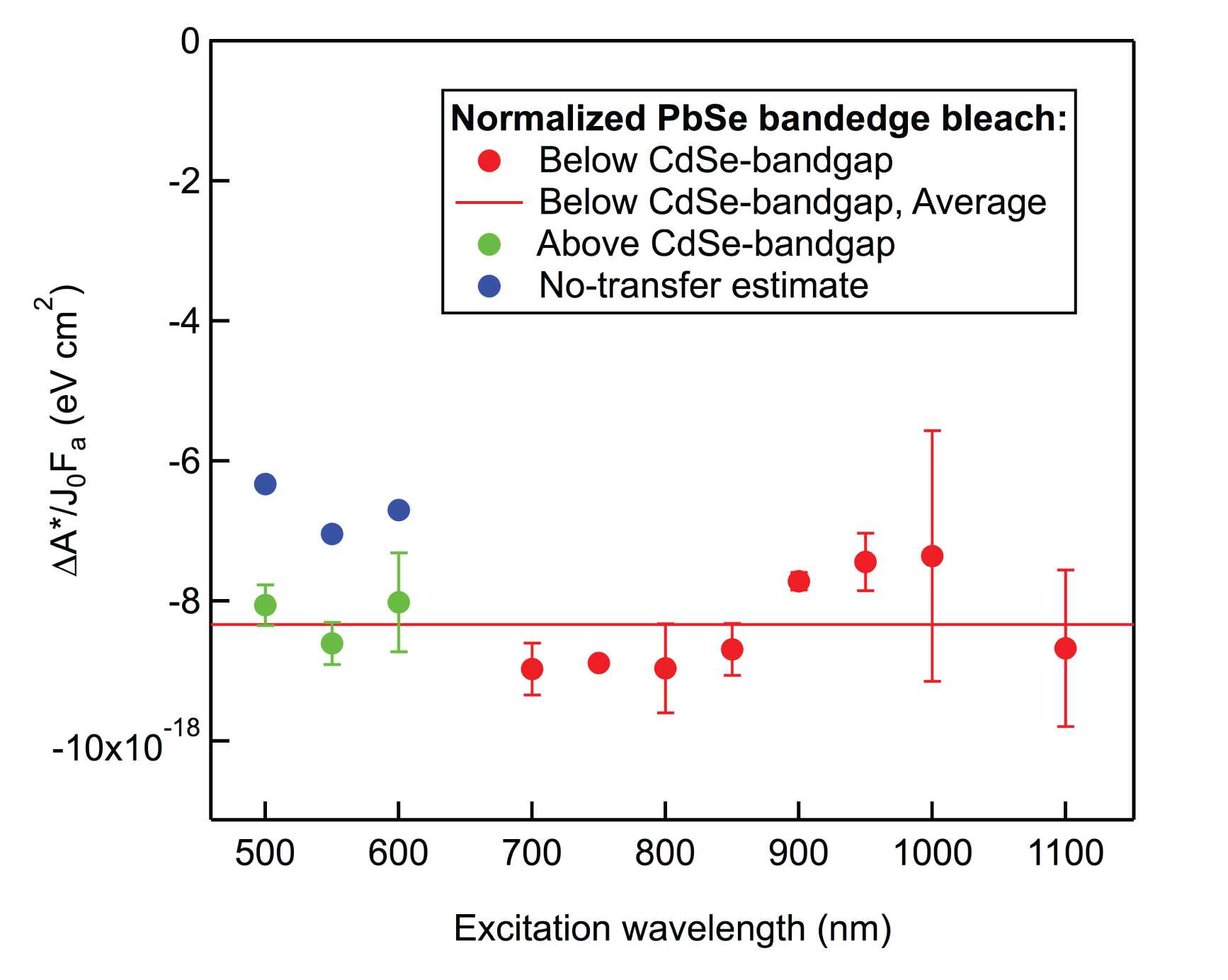


Supplementary Figure 7. Normalized PbSe TA bleach as a function of excitation wavelength. The red dots indicate the value of PbSe bleach for below CdSe-bandgap excitation, while the green dots show the PbSe bleach for above CdSe-bandgap excitation. Comparing the average value of the PbSe bleach for below CdSe-bandgap excitation (red line) with the green dot values indicates that the two excitation regimes produce the same amount of PbSe bleach, within experimental errors. Furthermore, estimating the reduction of PbSe bleach in the case of negligible electron transfer (blue dots) indicates that the constant PbSe bleach value is associated with efficient transfer from CdSe to PbSe QDs. Error bars are obtained repeating the measurement with the same excitation wavelength multiple times and calculating the standard deviation of the normalized TA bleach.

**Supplementary Note 6: Fitting method for TA measurements**

To solve the spectral overlap between the TA features of the two QDs in the visible range we fitted each TA spectrum with the function:

$$f\left( \lambda, t \right)= A_{g}(t)\exp\left[ -\frac{\left( \lambda-\lambda_{g} \right)^{2}}{2\cdot{\sigma_{g}}^{2}} \right]+A_{sh}(t) \left\{ \exp\left[ -\frac{\left( \lambda-\lambda_{g} \right)^{2}}{2\cdot{\sigma_{g}}^{2}} \right]- \exp\left[ -\frac{\left( \lambda-\lambda_{g}-\Delta\lambda_{sh} \right)^{2}}{2\cdot{\sigma_{g}}^{2}} \right] \right\}+A_{bg}(t) \left( \lambda-\lambda_{bg}(t) \right)$$

The first term, a Gaussian peak, accounts for the bleach of the Gaussian-shaped CdSe 1S feature. The second term describes the TA signal produced by a shift of the linear absorption associated with electrostatic interaction: it’s obtained by subtracting the Gaussian feature of the unperturbed CdSe 1S absorption by the same Gaussian feature shifted by $\Delta\lambda_{sh}.$ The last term is a line, empirically describing the shape of the slowly varying PbSe induced absorption background in a small wavelength range around the CdSe 1S feature. Of the 7 fit parameters, 3 are held constant: $\lambda_{g}$ and $\sigma_{g}$ are extracted from a fit of the CdSe 1S linear absorption in a CdSe-only film; $\Delta\lambda_{sh}$ is fixed at 5 nm, chosen to be a reasonable value for a Stark-shift of the CdSe 1S transition (more details on the choice for $\Delta\lambda_{sh}$ are provided in the following section). Thus the TA signal originating from the CdSe QDs is fitted changing the amplitudes of two fixed-shape features, while the line describing the PbSe background is left unconstrained.

The bleach amplitude $A_{g}(t)$, extracted from the fit, is multiplied by $\sqrt{2\pi} \sigma_{g}$ to obtain the energy integrated bleach amplitude ${A^{*}}_{g}(t)$, whose value divided by the absorbed photon fluence is shown in Figure 4a for different excitation energy.

**Supplementary Note 7: Effect of the choice of** $\boldsymbol{\Delta}\boldsymbol{\lambda}_{\boldsymbol{sh}}$ **on the TA fit**


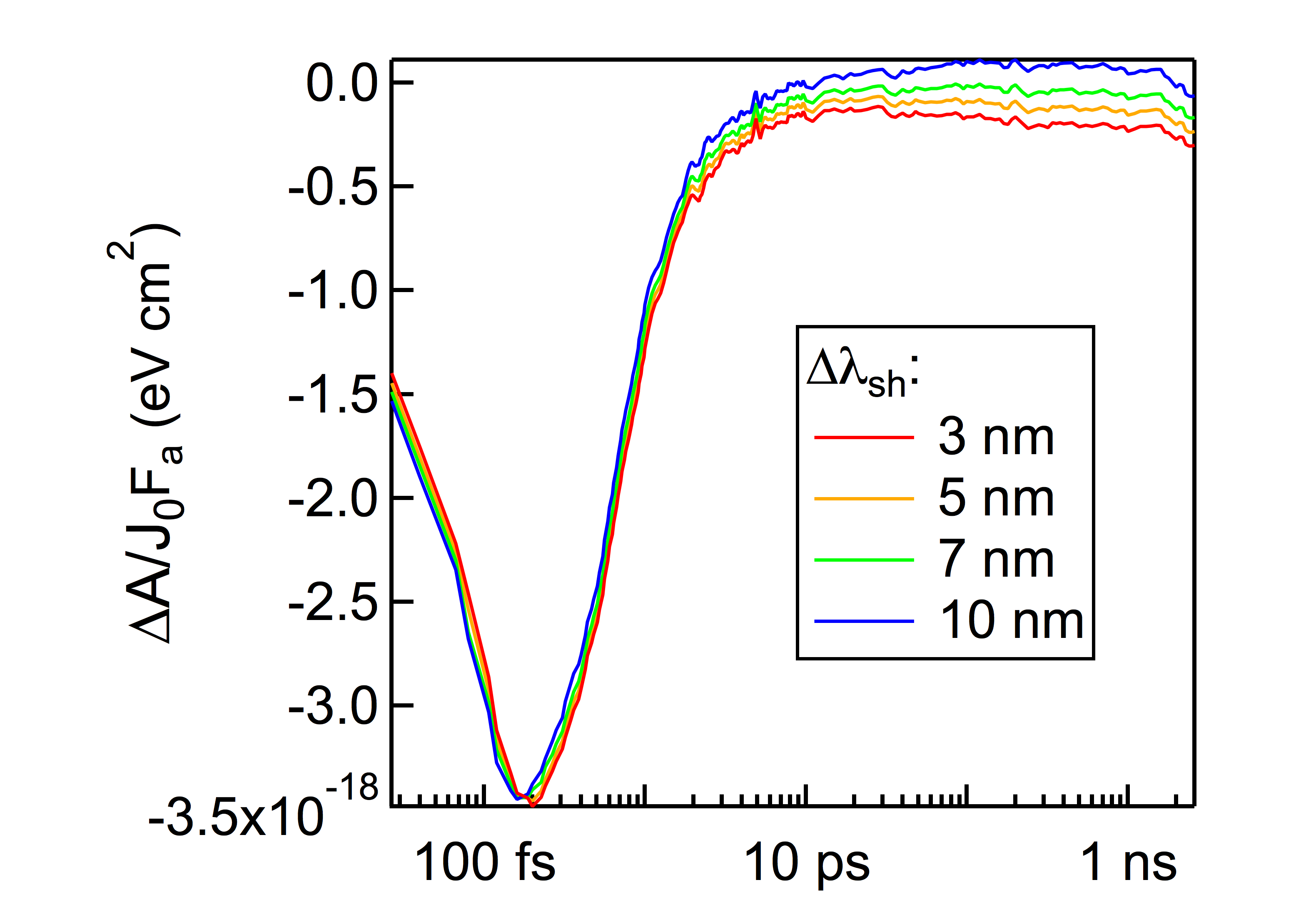


Supplementary Figure 8. Influence of $\boldsymbol{\Delta}\boldsymbol{\lambda}_{\boldsymbol{sh}}$ on the fit of the CdSe 1S bleach. Time-dependence of CdSe rescaled bleach obtained fitting a TA measurement with different values of the $\boldsymbol{\Delta}\boldsymbol{\lambda}_{\boldsymbol{sh}}$ parameter. The TA measurement was performed exciting at 700 nm, with a fluence of 1.05*∙*10^13^ photons/cm^2^ ·pulse.

The $\Delta\lambda_{sh}$ parameter of the TA fit was held at a constant value of 5 nm, corresponding to a 17 meV redshift of the CdSe absorption spectrum. Similar shift values were obtained in electric-field dependent PL measurements on CdSe QDs under a ~100 kV/cm electric field^3^. Supplementary Figure 8 shows the CdSe bleach rescaled by the absorbed photon fluence, obtained fitting the same TA measurement (700 nm excitation, 1.05***∙***10^13^ photons/cm^2^ **·** pulse) with $\Delta\lambda_{sh}$ values between 3 and 10 nm. While the choice for $\Delta\lambda_{sh}$ affects appreciably the bleach estimate in the 10 ps – 3 ns range, the peak value of the bleach remains largely unaffected. Thus the HET efficiency, based on the peak value of the bleach, can be safely estimated considering any $\Delta\lambda_{sh}$value in the 3 – 10 nm range.

**Supplementary Note 8: Response of CdSe QD film in TA measurements with below-bandgap excitation**

As mentioned in the main text, exciting a CdSe QD film below the onset of its linear absorption still results in bleaching of the CdSe bandedge absorption, which is however negligible compared to the magnitude of bleach arising in the HJ film excited at the same wavelength with comparable power (Figure 2f).


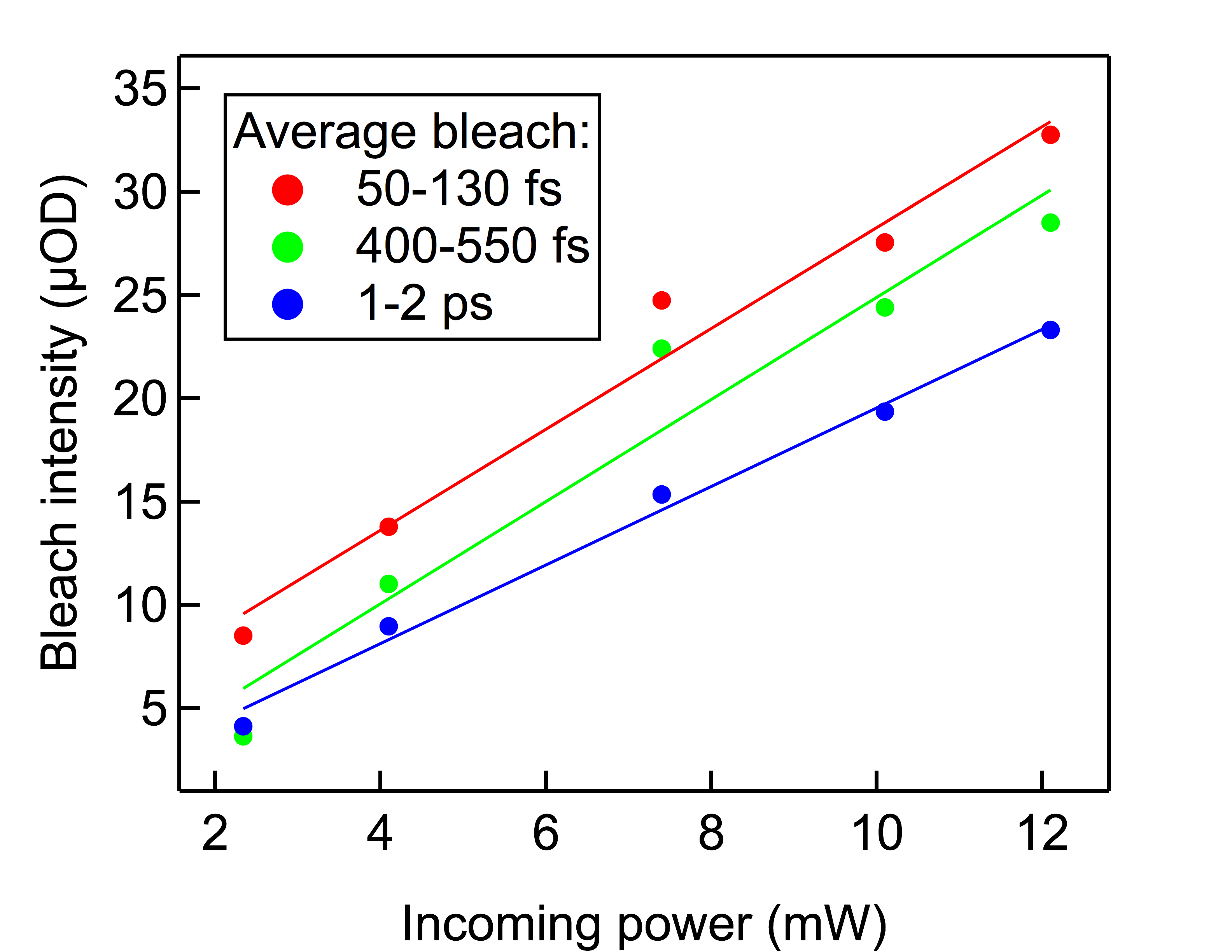


Supplementary Figure 9. Power dependence of below-bandgap excitation response in a CdSe QD film. Exciting a CdSe QD film with 700 nm light, a small bleach of the CdSe 1S feature develops. The intensity of the bleach signal scales linearly with the incoming power.

We characterized the behavior of the CdSe QD film upon below-bandgap excitation, measuring the power-dependence of the bleach resulting upon 700 nm excitation. Supplementary Figure 9 shows the power-dependence of the bleach signal, averaged over three different time intervals, showing a linear dependence.


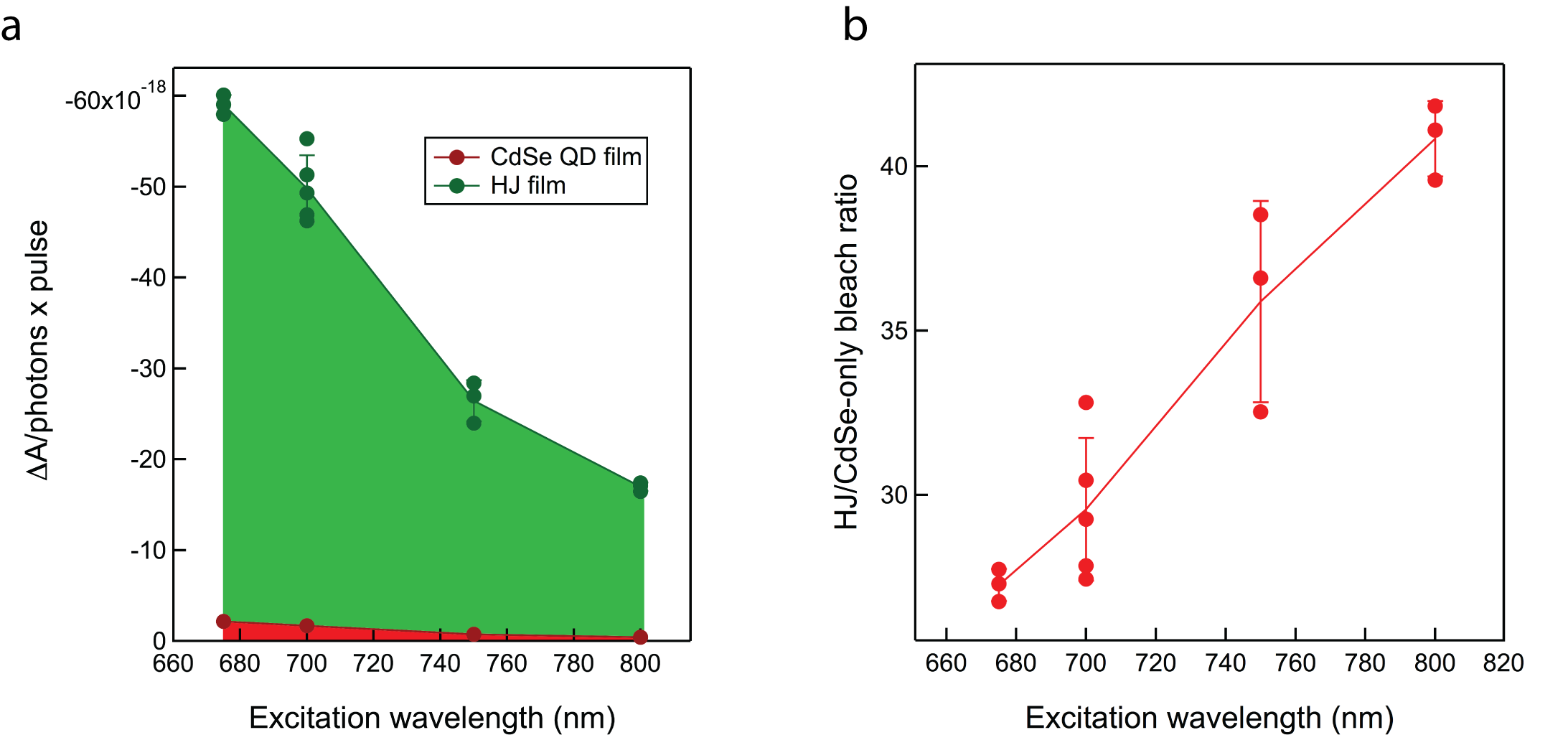


Supplementary Figure 10. Estimate of the contribution of direct CdSe excitation to the CdSe 1S bleach. a, bleach per incoming photon per pulse in the HJ and CdSe QD film, as a function of excitation wavelength. The graph makes clear that the bleach arising from CdSe QDs alone does not contribute significantly to the signal observed in the HJ film. b, ratio between the CdSe bleach amplitude observed in the HJ film and the same quantity measured in the CdSe QD film. Error bars represent the standard deviation of the ratios at each excitation wavelength, obtained from repeated measurements.

The presence of a bleach feature in the CdSe QD film for below-bandgap excitation is clearly arising from a different process than the one proposed for the HJ film, involving transfer of hot-electron from PbSe to CdSe QDs. Thus, is crucial for our interpretation of the CdSe TA bleach in the 675-1100 nm excitation range that the bleach observed in the CdSe QD film is negligible compared to the same quantity measured in the HJ film. We compared the amplitude of the bleach feature in the CdSe QD film and HJ film, for excitations in the 675-800 nm range. The bleach amplitudes were rescaled by the number of incoming photons in each pulse, exploiting the linearity of the bleach with the incoming power, in order to compare measurements with different powers. Supplementary Figure 10a shows the bleach per incoming photon in the two films. Supplementary Figure 10b shows the ratio between the bleach amplitude in the HJ film and in the CdSe QD film, indicating that the HJ film signal remains more than 20 times higher than the signal observed in the CdSe QD film.

**Supplementary Note 9: Determination of the bleach cross-section of CdSe QDs**

In order to quantify the efficiency of the transfer process, we need to estimate the bleach induced in a CdSe QD by a unit area density of electrons; i.e. the bleach cross-section $\sigma_{b}$. As reported by Boehme *et al.*^2^, the energy integrated differential absorption of the CdSe 1S feature can be related to the area-density of electrons $N_{e}$, by

$${\Delta A}^{*}=N_{e}\frac{\sigma_{b}}{\text{ln}(10)}$$

where $\sigma^{*}$ is an energy integrated bleach cross-section and $F_{a}J_{0}$ is the absorbed photon fluence. In a CdSe QD-film, directly excited and in absence of carrier multiplication or significant Auger recombination during the excitation pulse, each absorbed photon leads to the creation of one exciton. Thus, directly after photoexcitation, $\text{N}_{e}=F_{a}J_{0}$ and the bleach cross-section can be determined as:

$$\sigma_{b}= \frac{{\Delta A}^{*}(\text{t}=0, \text{CdSe-only})}{F_{a}J_{0}} \text{ln}(10)$$

**Supplementary Note 10: Rate-equation model for the fitting of CdSe bleach dynamics**


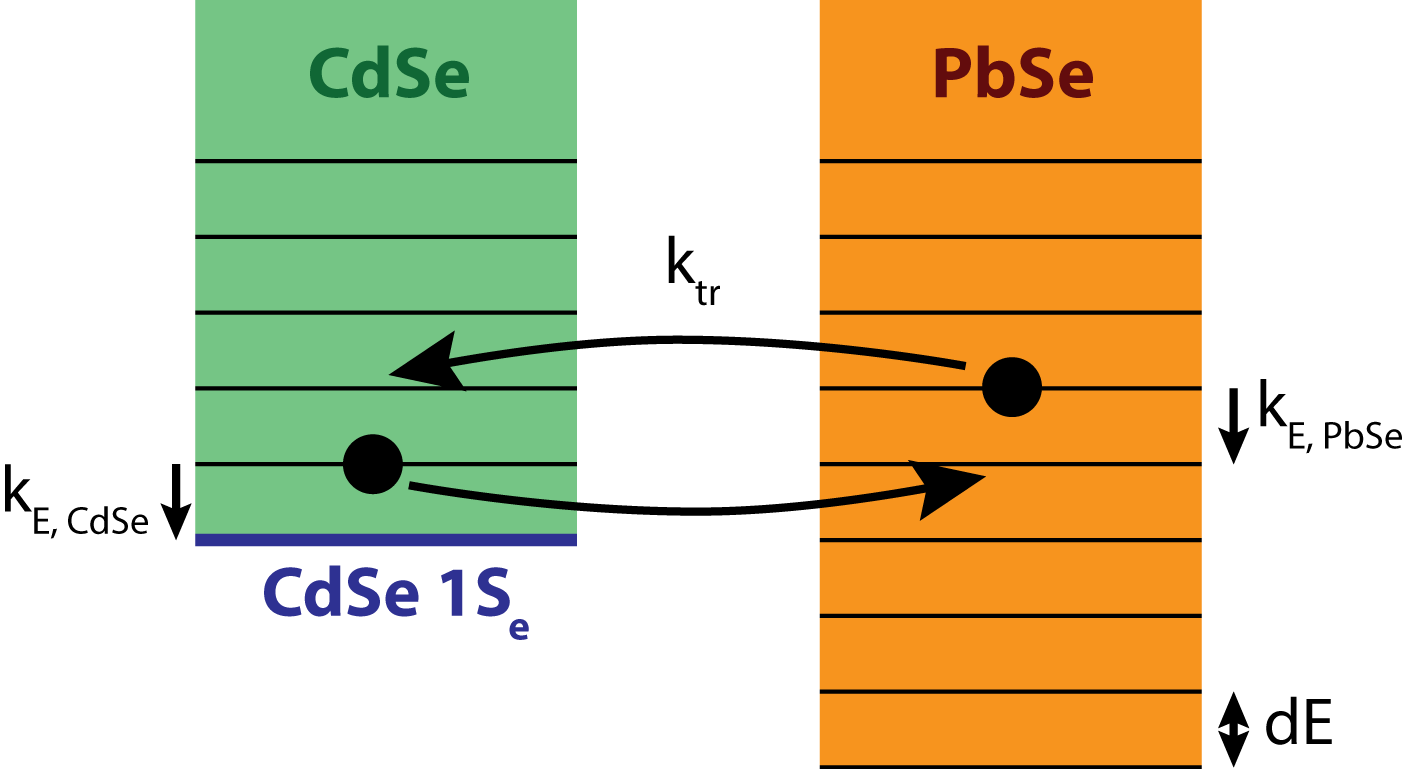


Supplementary Figure 11. Schematics of the energy structure in the rate-equation model. The conduction bands of the two materials are represented via two sets of energy states, separated by an arbitrarily chosen energy spacing dE. The two cooling rates ($\boldsymbol{k}_{\mathbf{E, CdSe}}$ and $\boldsymbol{k}_{\mathbf{E, PbSe}}$) and the transfer rate ($\boldsymbol{k}_{\boldsymbol{tr}}$) are constant in energy.

In order to rationalize the dynamics of the CdSe bleach observed in the TA measurements, we consider HET to be in competition with cooling and back-transfer of injected electrons to PbSe QDs, and employ a rate-equation model to qualitatively describe the process. The conduction bands of the two materials are modeled by two sets of electron levels, uniformly spaced in energy and displaced by a fixed conduction band offset, determined spectro-electrochemically (Supplementary Note 3). The energy spacing between levels, $\mathrm{dE}$, is taken as small as possible, to simulate a continuous density of states. This choice is motivated by the lack of detailed information on the position of energy levels in the coupled QD film. The rate of population change of an electron level $N_{x}$ (x = CdSe or PbSe) is determined by:

$$\frac{\text{d}N_{x}}{\text{d}t}\left( t, E \right)=k_{tr}\left( N_{y}\left( t,E \right)-N_{x}\left( t,E \right) \right)\Theta\left( E-E_{CdSe,CB} \right)+k_{E, x}\left( N_{x}\left( t,E+\text{d}E \right)-N_{x}(t,E) \right) \Theta\left( E-E_{x,CB} \right)$$

where $k_{tr}$ and $k_{E,x}$ are, respectively, the rate of transfer between the two QDs and a transition rate to the following lower energy state in the same QDs, while $\text{d}E$ indicates the chosen energy step. $\Theta$ indicates the Heaviside function, stopping the transfer between the two QDs for states below the lowest CdSe state and stopping the cooling at the conduction band edge of each material. The transfer rate and the cooling rate are assumed to be independent of electron energy and the transfer rate is assumed to be equal in both directions, in order to keep the model as simple as possible.

The simulation is initialized with the electron density completely localized on a single PbSe level at energy E_max_, determined considering a symmetric division of the photon excess energy between electron and hole. As the simulation evolves, the electron density starts spreading to lower energies and transferring to the CdSe QD component. At each time t_i_, the electron density N(t_i_, E_i_) is equal to the probability of finding an electron in E_i_ at time t­­_I_, given an initial excitation at the energy E_max_.

The model was used to fit the time-dependence of the fractional CdSe 1S_e­_ population, determined experimentally, using the transfer rate and the two materials cooling rates as fitting parameters (Supplementary Figure 11). The rate $k_{tr}$ sets the cooling dynamics in each of the two energy continua, and, as such, depends on the choice of the energy spacing between levels. We can relate the value of $k_{tr}$ to the experimentally determined energy loss rate, $\gamma_{E}=k_{tr}\cdot\text{dE}$.


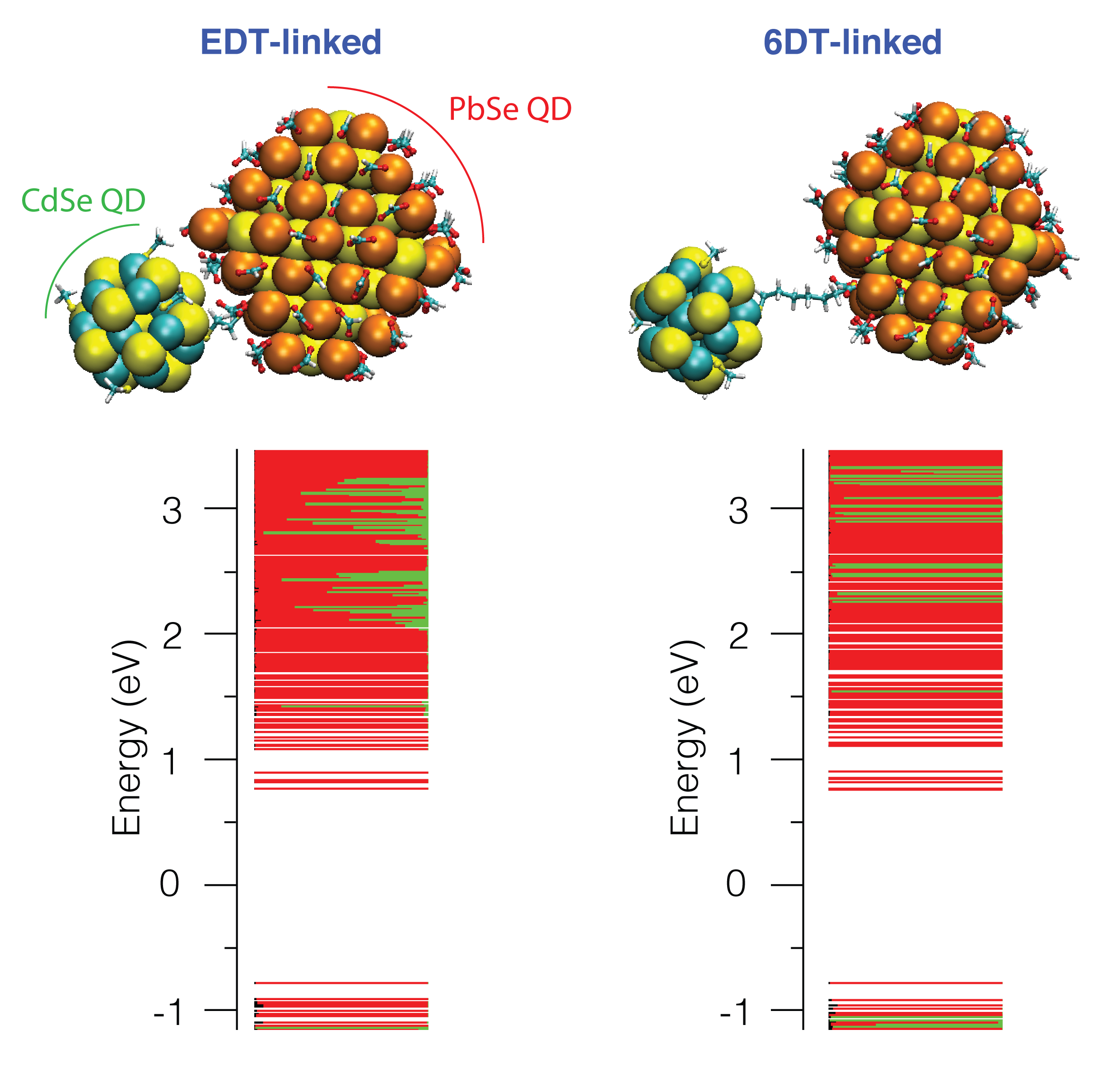


Supplementary Figure 12. Influence of ligand length on the electronic structure. Conduction band electronic structure of a coupled CdSe QD – PbSe QD system (see Methods) for two different bridging molecules: ethanedithiol (EDT) and hexanedithiol (6DT). Each line corresponds to a molecular orbital (MO), and the color of the line represents the contribution of each fragment of the simulation to the MO: the PbSe fragment is depicted in red, CdSe in green, and the organic bridge in black. It can be seen that for the EDT linked system, most of the MOs with a contribution from the CdSe fragment have a significant contribution from the PbSe fragment, resulting in a high degree of electron delocalization. Conversely, for the 6DT-linked system, MOs are mostly localized on one of the two QDs.


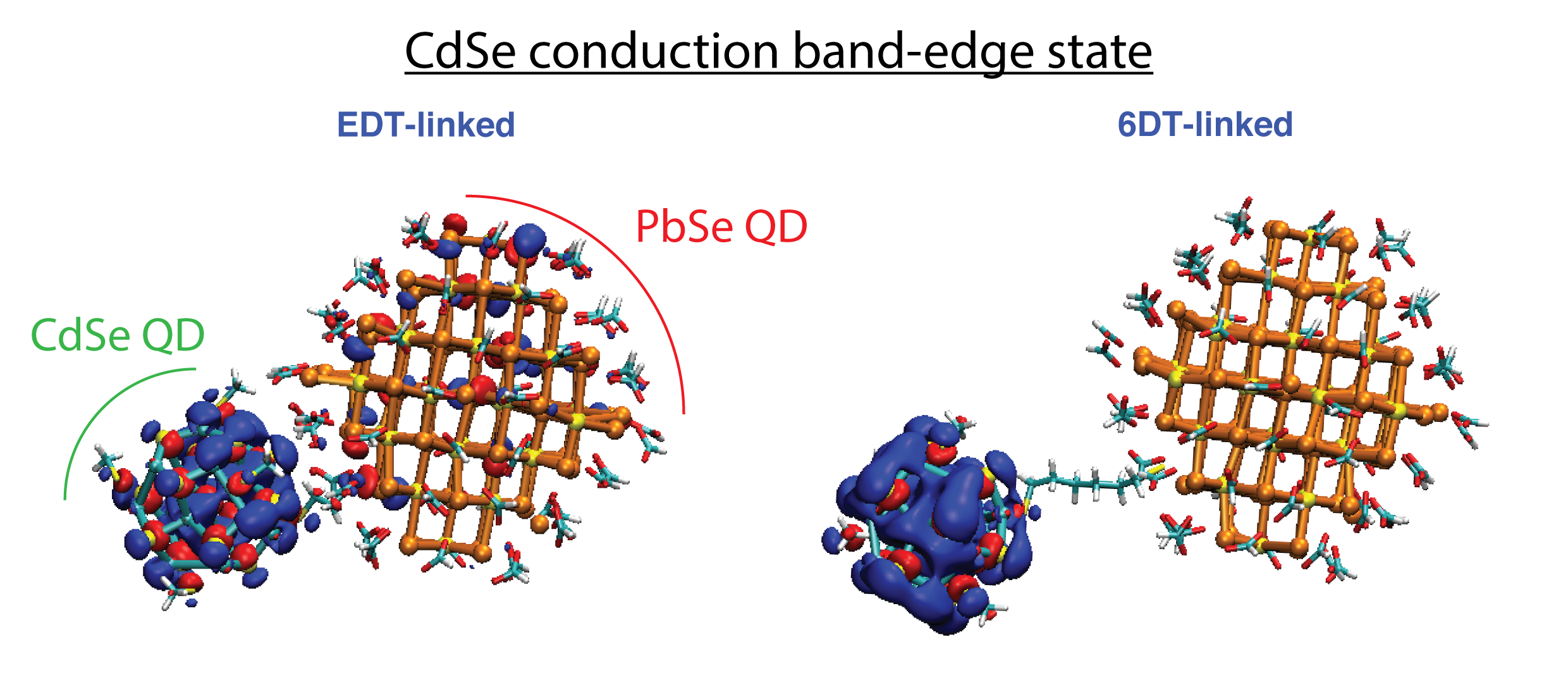


Supplementary Figure 13. Influence of ligand length on electron delocalization. MO plot for the lowest energy MO with a significant contribution from the CdSe fragment. The EDT-linked system shows delocalization of the MO on the PbSe QD, while the 6DT-linked MO remains localized on the CdSe QD.

**Supplementary References**

1. Boehme, S. C.*, et al.* Electrochemical charging of CdSe quantum dot films: dependence on void size and counterion proximity. *ACS Nano* **7,** 2500-2508 (2013).

2. Boehme, S. C.*, et al.* Electrochemical Control over Photoinduced Electron Transfer and Trapping in CdSe-CdTe Quantum-Dot Solids. *ACS Nano* **8,** 7067-7077 (2014).

3. Empedocles, S. A. & Bawendi, M. G. Quantum-confined stark effect in single CdSe nanocrystallite quantum dots. *Science* **278,** 2114-2117 (1997).

4. Sandeep, C. S. S.*, et al.* Epitaxially Connected PbSe Quantum-Dot Films: Controlled Neck Formation and Optoelectronic Properties. *ACS Nano* **8,** 11499-11511 (2014).

5. Gao, Y.*, et al.* Photoconductivity of PbSe Quantum-Dot Solids: Dependence on Ligand Anchor Group and Length. *ACS Nano* **6,** 9606-9614 (2012).
