# Supplementary material for: Hot-electron transfer in quantum-dot heterojunction films
Source: Nat Commun. 2018 Jun 13;9:2310. doi: 10.1038/s41467-018-04623-9 (PMC5998019; doi:10.1038/s41467-018-04623-9)
Supplement: Supplementary file 2 — Peer Review File [file 41467_2018_4623_MOESM2_ESM.pdf]

Reviewer #1 (Remarks to the Author):

This paper reports on the migration of electrons from one quantum dot (QD) to another. The specific focus is on the transfer of hot electrons, meaning electron transfer prior to exciton cooling. The process itself is of basic science interest. And the process may be useful in solar cell and related applications. In short, the topic and context is highly suitable for this Journal. The quality of the science is excellent. The data and the writing are great, and a pleasure to read. The control experiments are convincing. My only issue is that the basic spectroscopy and exciton dynamics deserve to be more rigorously discussed. Details below.

1. P.2 line 50 has text discussing exciton cooling processes. The authors discuss the phonon bottleneck and experimental investigations therof. But this entire discussion is done at the level of 1998 investigations, largely by Klimov and Guyot-Sionnest. There has been much advance in our understanding based upon state-resolved exciton dynamics which gives a far more detailed understanding than those prior works. In particular, the phonon bottleneck and that discussion was merely based upon too simple a theory. A more comprehensive theory deals with several relaxation channels that are now precisely measured and corroborate more comprehensive theory: Phys. Rev. Lett., 98, 177403 (2007); Phys. Rev. B., 78, 245311 (2007); J. Phys. Chem. C, 115, 22809 (2011).

2. The following paragraphs on p2 and p3 are well done, well conceived, and nicely illustrate the idea here. This is a great idea with great execution.

3. The text on p3 line 115 continues the discussion of how to analyze the transient absorption (TA) signals. Again, this could be better done. There is Ground State Bleach, Stimulated Emission, Excited State Absorption. And in the case of photoinduced absorptions these arise from excitation in to biexcitons and not a trapped carrier Stark effect. The latter was merely old language which is not up to date: J. Phys. Chem. C, 115, 22809 (2011); J. Phys. Chem. Lett, 3, 1182 (2012).

4. The authors, like others cited here discuss the process of hot electron transfer. What is missing is a more detailed discussion in light of the very well established physical chemistry of electron transfer in molecular systems. I suggest the author familiarize themselves with some of the classic literature on this topic so as to elevate the discussion. Here is one of many excellent such articles: Contemporary Issues in Electron Transfer Research, J. Phys. Chem., 1996, 100 (31), pp 13148–13168.

5. Once one is familiar with the salient processes of relaxation vs transfer, one can better discuss the phenomena. Nothing here is specific to nanocrystals, and even less so to the process of

charge transfer from nanocrystals. These are well studied phenomena. But this specific work does a great job of showing a classical effect in a new system with a great motivation. With that, the general idea is the competition kinetics between hot exciton cooling and hot exciton transfer. The transfer of charge could be an electron or hole. But the ideas are general. Cooling takes place on a ~ 1 ps timescale so perhaps transfer takes place on a ~10 ps timescale to give some yield of 0.1. These are merely examples, but the idea remains. Indeed this idea has been explored in detail for hot exciton trapping to the surface of the NC. Surface trapping is not the same as charge transfer that the authors do here, but the ideas are the same. The following papers all give detailed discussion and measurement of hot exciton surface trapping: J. Chem. Phys., 129, 084701 (2008); Nano Lett. 10, 3062 (2010); J. Chem. Phys., 134, 094706 (2011). In addition to TA measurements of the very similar process, one can have steady state measurements of the quantum yield spectrum for PL: J. Phys. Chem. C, 118, 7730 (2014). The point is that there is a large body of work that is highly relevant and supports the results here. It is important to note that the idea of hot carrier electron transfer is not something new that was discovered in this report. But that this report shows this classical effect in a compelling way.

6. The authors use fluence to report on excitation conditions. But it would be useful to estimate the mean exciton occupancy <N> in the standard methods.

7. The data in Fig2e is spectacular. Very compelling and very rich. P5 line 180 discusses these data. The main features are the CdSe 1S bleaching buildup and recovery, and also the photoinduced absorptions. As noted above, the state-resolved exciton dynamics in CdSe model systems enable a much more detailed discussion of the signals and what processes give rise to them. For example, how fast is the buildup compared to CdSe 1S pump? It should be IRF limited there, but with a buildup time given by the hot electron transfer time. The depletion is the back electron transfer (note this mirrors the discussion of photoinduced ET in the review by Barbara et al). with b-ET, one has buildup of charges on PbSe and these give the photoinduced absorptions shown here. Not a Stark effect. Absorption into biexcitons. The detailed works on multiexcitons explain the signals in detail: J. Phys. Chem. C, 115, 22809 (2011); J. Phys. Chem. Lett, 3, 1182 (2012); J. Chem. Phys., 134, 094706 (2011). The worked up data in Fig3 here reproduces the earlier works.

8. The data on effiency is very nice too. But I would like to see data with CdSe 1S pump. The idea is to see the buildup time. Is there some energy dependence to the buildup time?

9. Can the authors suggest what goes into the efficiency? One might think the efficiency is just given by competition kinetics of cooling vs transfer. And both will have an energy dependence. But this is what happens for charge trapping at the surface. The transfer of charge to the other NC may be something different.

Reviewer #2 (Remarks to the Author):

In this manuscript, the author observed bleaching of the 1S transition in CdSe QDs through transient absorption spectroscopy even with sub-bandgap excitation when assembled with PbSe QDs to form type-I band alignment. It was attributed to hot-electron transfer between the two QD components in the HJ film. The hot-electron transfer efficiency was quantified by integrating the differential absorbance at the 1S transition of CdSe QDs and shown to have excitation energy dependence. By fitting the change of transfer efficiency with time, the author was also able to extract the electron transfer rate, energy loss rate, etc. during the process. All the experimental observations were consistent with the result obtained from the DFT modeling.

I think this manuscript is clearly written with conclusions significant enough for publishing with minor revisions:

1. How monodisperse are the CdSe and PbSe QDs? Based on the UV-Vis absorption spectra, the exciton peak of PbSe QDs is noticeably broader. How does the charge/energy transfer within different-sized PbSe/CdSe QDs compete with the hot-electron transfer across the two QD species?

2. How many electrons are generated in PbSe QDs and transferred to CdSe QDs under the experimental fluences? The increase of electrons in CdSe QDs from hot-electron transfer can facilitate Auger recombination, which can be another cause of the fast decay of the bleach at CdSe 1S energy besides back-transfer.

3. In the middle of page 5, the author tries to verify the back-transfer by exciting CdSe and counting bleach at PbSe 1S transition, which I doubt its sufficiency because it can be excitons and/or energy besides just electron transfer from excited CdSe to PbSe QDs.

4. Line 271, the equation for bleach cross-section is miswritten.

Addressing the concerns of reviewer 1:

Reviewer comments:
This paper reports on the migration of electrons from one quantum dot (QD) to another. The specific focus is on the transfer of hot electrons, meaning electron transfer prior to exciton cooling. The process itself is of basic science interest. And the process may be useful in solar cell and related applications. In short, the topic and context is highly suitable for this Journal. The quality of the science is excellent. The data and the writing are great, and a pleasure to read. The control experiments are convincing. My only issue is that the basic spectroscopy and exciton dynamics deserve to be more rigorously discussed. Details below.

Reply: We thank the reviewer for this favorable evaluation. As discussed below we have used his suggestions to strengthen the discussion on the spectroscopy and exciton dynamic.

1. P.2 line 50 has text discussing exciton cooling processes. The authors discuss the phonon bottleneck and experimental investigations therof. But this entire discussion is done at the level of 1998 investigations, largely by Klimov and Guyot-Sionnest. There has been much advance in our understanding based upon state-resolved exciton dynamics which gives a far more detailed understanding than those prior works. In particular, the phonon bottleneck and that discussion was merely based upon too simple a theory. A more comprehensive theory deals with several relaxation channels that are now precisely measured and corroborate more comprehensive theory: Phys. Rev. Lett., 98, 177403 (2007); Phys. Rev. B., 78, 245311 (2007); J. Phys. Chem. C, 115, 22809 (2011).

Reply: We fully acknowledge the complexity of the phonon bottleneck effect, and note that we have studied this in great detail recently for PbSe QDs in solution (ACS Nano 10, p695 and ACS Nano 11, p6286). We have expressed this more strongly in the revised introduction.
We mentioned already in line 59 that experimental evidence on the presence of phonon bottlenecks remain scattered, and that high carrier relaxation rates are found for quantum dots. Following his remark, we expanded the comment on the phonon mentioning that to properly exploit this process one needs to take care of the possible surface related relaxation channels. We have included the first and third citation suggested by the reviewer in the manuscript.

2. The following paragraphs on p2 and p3 are well done, well conceived, and nicely illustrate the idea here. This is a great idea with great execution.

Reply: We thank the reviewer for this supportive comment!

3. The text on p3 line 115 continues the discussion of how to analyze the transient absorption (TA) signals. Again, this could be better done. There is Ground State Bleach, Stimulated Emission, Excited State Absorption. And in the case of photoinduced absorptions these arise from excitation in to biexcitons and not a trapped carrier Stark effect. The latter was merely old language which is not up to date: J. Phys. Chem. C, 115, 22809 (2011); J. Phys. Chem. Lett, 3, 1182 (2012).

Reply: We acknowledge the reviewer's remarks on the nomenclature of the origin of the induced absorption features observed in TA, stressing the role of biexcitonic effects over a nomenclature suggesting a purely electrostatic picture. Thus we changed the reference to a "Coulomb shift" in the text, changing them into a "biexciton shift", where appropriate (e.g. line 149 and 220 of the revised manuscript), including the references suggested by the reviewer. At the same time we remark that spectral shifts are not

always due to biexciton effects and some of the shifts we observe at longer delay times are induced by trapped charges. Here, we argue, the term Coulomb shift is more appropriate.

4. The authors, like others cited here discuss the process of hot electron transfer. What is missing is a more detailed discussion in light of the very well established physical chemistry of electron transfer in molecular systems. I suggest the author familiarize themselves with some of the classic literature on this topic so as to elevate the discussion. Here is one of many excellent such articles: Contemporary Issues in Electron Transfer Research, J. Phys. Chem., 1996, 100 (31), pp 13148–13168.

We thank the reviewer for the suggested review, which we have studied upon his suggestion. We would argue we are quite familiar with electron transfer in molecular systems. Some of us have many publications on the matter, including for instance the review *Int. Rev. Phys. Chem.* **2008**, *27*, 87-138.

5. Once one is familiar with the salient processes of relaxation vs transfer, one can better discuss the phenomena. Nothing here is specific to nanocrystals, and even less so to the process of charge transfer from nanocrystals. These are well studied phenomena. But this specific work does a great job of showing a classical effect in a new system with a great motivation. With that, the general idea is the competition kinetics between hot exciton cooling and hot exciton transfer. The transfer of charge could be an electron or hole. But the ideas are general. Cooling takes place on a ~ 1 ps timescale so perhaps transfer takes place on a ~10 ps timescale to give some yield of 0.1. These are merely examples, but the idea remains. Indeed this idea has been explored in detail for hot exciton trapping to the surface of the NC. Surface trapping is not the same as charge transfer that the authors do here, but the ideas are the same. The following papers all give detailed discussion and measurement of hot exciton surface trapping: J. Chem. Phys., 129, 084701 (2008); Nano Lett. 10, 3062 (2010); J. Chem. Phys., 134, 094706 (2011). In addition to TA measurements of the very similar process, one can have steady state measurements of the quantum yield spectrum for PL: J. Phys. Chem. C, 118, 7730 (2014). The point is that there is a large body of work that is highly relevant and supports the results here. It is important to note that the idea of hot carrier electron transfer is not something new that was discovered in this report. But that this report shows this classical effect in a compelling way.

Reply: We fully agree that HET is not specific to QDs and admit that this possibility was not mentioned explicitly in the manuscript. In the revised manuscrtipt we included a general statement about the occurrence of HET (see line 64 of revised manuscript), while expanding our literature citations on the reports of HET towards localized surface states, including the first and third reference suggested by the reviewer.

6. The authors use fluence to report on excitation conditions. But it would be useful to estimate the mean exciton occupancy <N> in the standard methods.

Reply: Following the reviewer's suggestion we included for each reported TA measurement the estimation of the average exciton population in the PbSe QD component upon photoexcitation.

7. The data in Fig2e is spectacular. Very compelling and very rich. P5 line 180 discusses these data. The main features are the CdSe 1S bleaching buildup and recovery, and also the photoinduced absorptions. As noted above, the state-resolved exciton dynamics in CdSe model systems enable a much more detailed discussion of the signals and what processes give rise to them. For example, how fast is the buildup compared to CdSe 1S pump? It should be IRF limited there, but with a buildup time given by the hot electron transfer time.

The depletion is the back electron transfer (note this mirrors the discussion of photoinduced ET in the review by Barbara et al). with b-ET, one has buildup of charges on PbSe and these give the photoinduced absorptions shown here. Not a Stark effect. Absorption into biexcitons. The detailed works on multiexcitons explain the signals in detail: J. Phys. Chem. C, 115, 22809 (2011); J. Phys. Chem. Lett, 3, 1182 (2012); J. Chem. Phys., 134, 094706 (2011). The worked up data in Fig3 here reproduces the earlier works.

Following the suggestion from the reviewer we added in the Supporting Information a section displaying rise times of the CdSe bleach signal for different excitation energies, both below and above the threshold for direct excitation of CdSe QDs. Our data show a strong reduction of the rise time for direct CdSe excitation compared to PbSe excitation followed by HET, indicating that, in contrast to the IRF limited rise of the bleach signal upon CdSe 1S pump, injecting carriers in CdSe via HET requires additional time. The rise time of the HET CdSe bleach signal is determined both by the transfer rate and by cooling within the manifold of CdSe states, the latter preventing facile extraction of transfer rates from the rise time. We can exclude that the photoinduced absorptions arising after 100 ps from photoexcitation at the bandgap energy of CdSe QDs arise from charges located at the conduction band edge of PbSe QDs. In fact the derivative-like feature appears in a time-window in which the PbSe band-edge bleach decays, showing little decay well after the PbSe QD 1S absorption is fully recovered. Thus we interpreted this behavior in terms of electrons trapped at the interface between PbSe and CdSe QDs, and not as biexciton generation. With the information provided by the measurement we cannot establish whether the electrons are trapped at the CdSe or PbSe QD surface.

8. The data on effiency is very nice too. But I would like to see data with CdSe 1S pump. The idea is to see the buildup time. Is there some energy dependence to the buildup time?

Reply: For direct CdSe 1S pump the signal generated due to direct excitation largely overpowers the signal due to HET from PbSe QDs. In this case the rise time of the signal is simply the IRF, as addressed in the reply to point 7. The immage shown below shows the version of Figure 4a and 4b including the data for direct CdSe excitation as well. Efficiencies of initial electron injection in CdSe QDs are below unity even for direct CdSe excitation, due to the concurrent absorption from PbSe QDs.

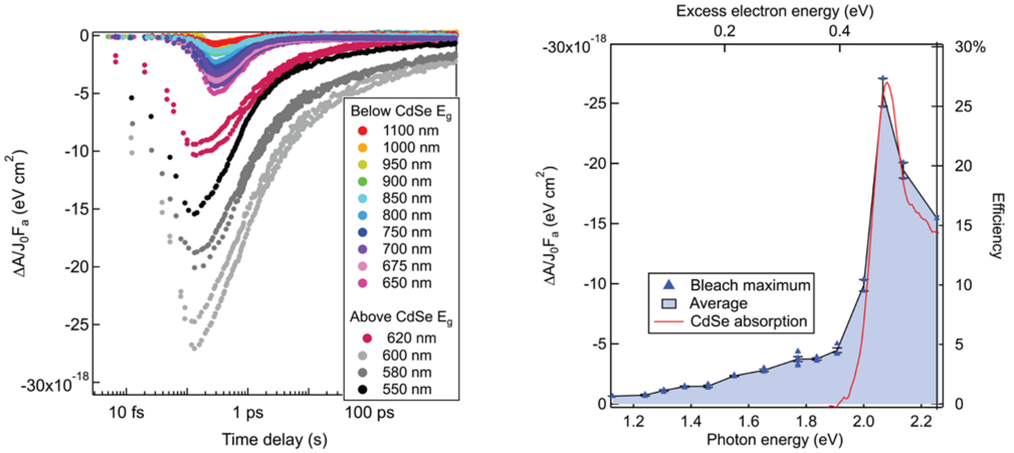

9. Can the authors suggest what goes into the efficiency? One might think the efficiency is just given by competition kinetics of cooling vs transfer. And both will have an energy dependence. But this is what happens for charge trapping at the surface. The transfer of charge to the other NC may be something different.

Reply: Indeed the efficiency trend can be explained in terms of a direct competition between electron cooling and electron transfer. Increasing the excitation energy increases the time-window for HET to take place, thus increasing the effective cooling time necessary for the electron to cross the threshold where transfer is no longer possible. At the same time higher electron energy leads both to a higher degree of electron delocalization, as indicated by the TDDFT results in the text, and to a higher number of final CdSe QD states, both effects increasing the rate of electron transfer. This picture is sufficient to justify the trend reported experimentally.

Addressing the concerns of reviewer 2:

Reviewer comments:
In this manuscript, the author observed bleaching of the 1S transition in CdSe QDs through transient absorption spectroscopy even with sub-bandgap excitation when assembled with PbSe QDs to form type-I band alignment. It was attributed to hot-electron transfer between the two QD components in the HJ film. The hot-electron transfer efficiency was quantified by integrating the differential absorbance at the 1S transition of CdSe QDs and shown to have excitation energy dependence. By fitting the change of transfer efficiency with time, the author was also able to extract the electron transfer rate, energy loss rate, etc. during the process. All the experimental observations were consistent with the result obtained from the DFT modeling.

I think this manuscript is clearly written with conclusions significant enough for publishing with minor revisions:

Reply: We thank the reviewer for this evaluation. We are happy that he/she finds the experiments convincing and the conclusions significant.

1. *How monodisperse are the CdSe and PbSe QDs? Based on the UV-Vis absorption spectra, the exciton peak of PbSe QDs is noticeably broader. How does the charge/energy transfer within different-sized PbSe/CdSe QDs compete with the hot-electron transfer across the two QD species?*

Reply: The width of the PbSe QDs 1S absorption is indeed significantly broader than for the CdSe QDs. This is due in part to the large influence of small size differences on the strongly confined PbSe QDs, but is also affected by the larger homogeneous linewidth of the PbSe QD 1S feature. Measuring the absorption spectrum of the 2.3 nm PbSe QDs in solution we obtained a FWHM of 124 meV, comparing favorably with FWHM values reported by Campos *et al.* (DOI: 10.1021/jacs.6b11021) for similar sizes of PbSe QDs. Furthermore upon film fabrication QD coupling and change in the dielectric environment further broaden the QD spectral features (see e.g. ACS Nano, 8, p11499, DOI: 10.1021/nn504679k). We expect some effect of the PbSe energy disorder on the electron transfer, namely that the cooled down electrons would preferentially hop to larger QD sites, having the lower 1Se energy. However for hot electrons the presence of a high density of electron states in PbSe QDs will smooth the energetic differences between different sizes of PbSe QDs, as quantum confinement plays a smaller role on higher electronic states.
Our analysis technique doesn't allow us to quantify the rate of electron or exciton hopping between QDs of the same species. However one could expect similar efficiency for the electron transfer between two PbSe QDs, possibly higher on account of the higher density of states at a fixed energy in PbSe QDs compared to CdSe QDs, increasing the rate of transfer. Following this interpretation, hot electron transfer occurs throughout the bi-component film, both across the PbSe homo-interface and across the hetero-interface. Potential PbSe-PbSe isoenergetic charge or exciton transfer will not influence the rate of HET to CdSe QDs.

2. *How many electrons are generated in PbSe QDs and transferred to CdSe QDs under the experimental fluences? The increase of electrons in CdSe QDs from hot-electron transfer can facilitate Auger recombination, which can be another cause of the fast decay of the bleach at CdSe 1S energy besides back-transfer.*

Reply: The observed decay of the CdSe QD bleach is independent from photon fluence. The fact that the fast decay remains for excitation conditions generating less than 0.1

excitons per PbSe QD, combined with the ~5% yield of the transfer process, suggest that the situation involving two transferred electrons in a CdSe QD is negligible. Furthermore holes are energetically restricted from transferring to CdSe QDs by the valence band offset between the two materials, which should decrease significantly the impact of Auger recombination on the bleach induced by the transferred electrons.

3. *In the middle of page 5, the author tries to verify the back-transfer by exciting CdSe and counting bleach at PbSe 1S transition, which I doubt its sufficiency because it can be excitons and/or energy besides just electron transfer from excited CdSe to PbSe QDs.*

We thank the reviewer for this comment. In quantum dot samples typical FRET lifetime span the 100ps to 1ns range (see Appl. Phys. Lett. **84**, 2904 (2004); doi: 10.1063/1.1702136). Transfer of electrons out of the CdSe QD conduction band edge happens on a ~1ps to 10ps timescale, suggesting the behavior is associated with charge transfer rather than excitonic transfer. Furthermore, the constant bleach for absorbed photon across the whole excitation range, showed in Figure S7, suggests that all of the photoexcited electrons and holes reach PbSe quantum dots within the cooling time.

4. Line 271, the equation for bleach cross-section is miswritten.
We have verified that the equation is correct by deriving it again and confirming it with earlier work that we published (ACS Nano 8, 7067).

Reviewer #1 (Remarks to the Author):

The authors have well addressed the questions. This revision is suitable for publication.

Reviewer #1 (Remarks to the Author):

The authors have well addressed the questions. This revision is suitable for publication.

Response to reviewers comments:

Reviewer 1: “The authors have well addressed the questions. This revision is suitable for publication.”

The reviewers didn’t require further changes.

Response to the editor requests:

1 - “Nature Communications uses a transparent peer review system, where for manuscripts submitted from January 2016 we are publishing the reviewer comments to the authors and author rebuttal letters of our research articles online as a supplementary peer review file. Please let us know in the cover letter when submitting the final version of your manuscript if you wish to opt out of this scheme or not. If you are concerned about the release of confidential data, we do permit redactions in the interest of confidentiality. If you would like to remove such information from these files, then please let us know specifically what information you would like to have removed. Please note that we cannot incorporate redactions for other reasons. Reviewer names will be published in the peer review files if the reviewer comments to the authors are signed by the reviewer, or if reviewers explicitly agree to release their name. For more information, please refer to our FAQ page at: https://media.nature.com/full/nature-assets/ncomms/authors/ncomms-transparent-peer-review.pdf

Response to 1 – We agree to the publication of the reviewer comments and author rebuttal letters. We included the same statement in the cover letter.

2 - Your manuscript should comply with our policies and format requirements, detailed in our checklist for authors at: http://www.nature.com/article-assets/npg/ncomms/authors/ncomms_manuscript_checklist.pdf

Response to 2: we revised our manuscript to comply with Nature Communications policies and format.

3 - Please also review the changes in the attached copy of your manuscript, which has been edited for style, and address the comments and queries I have added. If using Word, please use the 'track changes' feature to make the process of accepting your manuscript more efficient.

Response to 3: the changes highlighted in the edited version of the manuscript were implemented, keeping track of changes with the “track changes” option in Word.

4 - Data availability statements and data citations policy: All Nature Communications manuscripts must include a section titled "Data Availability" at the end of the Methods section or main text (if no Methods). For more information on this policy, and a list of examples, please

see http://www.nature.com/authors/policies/data/data-availability-statements-data-citations.pdf In particular, the Data availability statement should include: - Accession codes for deposited data - Other unique identifiers (such as DOIs and hyperlinks for any other datasets) - At a minimum, a statement confirming that all relevant data are available from the authors - If applicable, a statement regarding data available with restrictions - If a dataset has a Digital Object Identifier (DOI) as its unique identifier, we strongly encourage including this in the Reference list and citing the dataset in the Data Availability Statement.

Response to 4 – Our manuscript contains a Data Availbility section, referring to a dataset hosted on a repository service. The DOI and hyperlink to the dataset are provided.

5 - DATA SOURCES: We strongly encourage authors to deposit all new data associated with the paper in a persistent repository where they can be freely and enduringly accessed. We recommend submitting the data to discipline-specific, community-recognized repositories, where possible and a list of recommended repositories is provided here: http://www.nature.com/sdata/policies/repositories If a community resource is unavailable, data can be submitted to generalist repositories such as figshare (https://figshare.com/) or Dryad Digital Repository (http://datadryad.org/). Please provide a unique identifier for the data (for example a DOI or a permanent URL) in the data availability statement, if possible. If the repository does not provide identifiers, we encourage authors to supply the search terms that will return the data. For data that have been obtained from publicly available sources, please provide a URL and the specific data product name in the data availability statement. Data with a DOI should be further cited in the methods reference section. Please refer to our data policies here: http://www.nature.com/authors/policies/availability.html

Response to 5 – Addressed in response 4.

6 - To ensure correct hyperlinking of the accession codes in your manuscript, please add the hyperlink or DOI in square brackets directly after the code throughout (for example, '5XRN [http://dx.doi.org/10.2210/pdb5XRN/pdb]', '1483958 [https://dx.doi.org/10.5517/ccdc.csd.cc1lt5m6]', 'SRP109982 [https://www.ncbi.nlm.nih.gov/sra/?term=SRP109982]' or 'NQLW00000000 [https://www.ncbi.nlm.nih.gov/assembly/GCA_002312845.1/]'). * Please check whether your manuscript or Supplementary Information contain third-party images, such as figures from the literature, stock photos, clip art or commercial satellite and map data. We strongly discourage the use or adaptation of previously published images, but if this is unavoidable, please request the necessary rights documentation to re-use such material from the relevant copyright holders and return this to us when you submit your revised manuscript. Additionally, please ensure that an updated editorial policy checklist that verifies compliance with all required editorial policies is completed and uploaded as a Related Manuscript file type with the revised article. Please note that this form is a dynamic 'smart pdf' and must therefore be downloaded

and completed in Adobe
Reader. https://www.nature.com/authors/policies/Policy.pdf

Response to 6 – We ensured the DOI of the dataset is contained in square brackets.

7 - We are committed to ensuring clarity and avoiding ambiguity in the mathematics in our papers. Consequently, please carefully check the mathematical terms throughout your manuscript (including labels on figures and figure captions) to ensure that it conforms strictly to the following guidelines. In mathematical terms, scalar variables (e.g. x, V, χ) should be typeset in italic, whereas multi-letter variables should be formatted without italic. Constants (e.g. ħ, G, c) should be typeset in italics (the only exceptions being e, i, π, which should be typeset without italic) and vectors (such as r, the wavevector k, or the magnetic field vector B) should be typeset in bold without italics. In contrast, subscripts and superscripts should only be italicized if they too are variables or constants. Those that are labels (such as the 'c' in the critical temperature, T_c, the 'F' in the Fermi energy, E_F, or the 'crit' in the critical current, I_crit) should be typeset in roman. Please also ensure the same convention is followed in figure labels, axes, and such. Additionally, to avoid doubt, unit dimensions should be expressed using negative integers (e.g. kg m^-1 s^-2 not kg/ms^2) or the word 'per'.

Response to 7 – We ensured mathematical formulas are expressed according to Nature Communications style format.

8 - Your paper will be accompanied by a two-sentence summary, of between 250-300 characters (including spaces), when it is published on our homepage. Could you please provide a draft summary in your cover letter which the editor will check, edit and approve. The first sentence should provide a brief context, whilst the second sentence should describe the current results, starting with “Here, the authors…”. Please take a look at recent examples on our homepage for guidance: http://www.nature.com/ncomms/

Response to 8 – We provided a short summary of our work in the Cover letter.
